# Concentration-Dependent Interlaminar Fracture Toughening in Carbon, Glass, and Hybrid Fiber-Reinforced Polymer Laminates via MWCNT Interlayers

**DOI:** 10.3390/polym18202515

**Published:** 2026-10-14

**Authors:** Shile Zhang, Ju Yang, Xiang Peng, Lvze Dai, Dongdong Chen, Guohua Zhu, Zhen Wang

**Affiliations:** 1 State Key Laboratory of Rail Transit Vehicle System, Southwest Jiaotong University, Chengdu 610031, China; 2 School of Automobile, Chang’an University, Xi’an 710064, China; 3 Chang’an Dublin International College of Transportation, Chang’an University, Xi’an 710018, China

**Keywords:** multi-walled carbon nanotubes, interlaminar toughening, carbon/glass hybrid laminates, Mode I and Mode II fracture, fracture-surface morphology

## Abstract

Fiber-reinforced polymer (FRP) laminates are prone to delamination because they lack continuous through-thickness reinforcement. Although multi-walled carbon nanotube (MWCNT) interlayers can improve interlaminar resistance, evidence has focused mainly on unidirectional carbon fiber-reinforced polymer (CFRP) and individual fracture modes. This study compared Mode I propagation and Mode II initiation toughness in CFRP, glass fiber-reinforced polymer (GFRP), and two carbon/glass hybrid laminates (HCRP and HGRP). Each system included unmodified (ORI), neat-epoxy interlayer (CON), and 0.1, 0.3, and 0.5 wt.% MWCNT conditions. The laminates were fabricated by N,N-dimethylformamide-assisted ultrasonication and hot pressing, and examined by ASTM D5528 double cantilever beam testing, ASTM D7905 end-notched flexure testing, and scanning electron microscopy. ORI and CON exhibited comparable toughness, indicating little contribution from the neat-epoxy interlayer alone. In all four systems, both toughness measures increased from 0.1 to 0.3 wt.% and decreased at 0.5 wt.%. At 0.3 wt.%, steady-state Mode I toughness increased by 31.2–51.2% and Mode II initiation toughness increased by 4.0–23.1% relative to CON. GFRP showed the highest absolute Mode I toughness, HCRP showed the largest Mode II improvement, and HGRP exhibited limited Mode II sensitivity. Matrix tearing, shear cusps, tortuous crack features, and distributed MWCNT pull-out at 0.3 wt.% contrasted with smoother regions, bundled or agglomerate-like structures, and cavities or pores at 0.5 wt.%. The effective MWCNT content therefore reflects a balance between nanotube-mediated energy dissipation and defect formation, modulated by fiber architecture and fracture mode.

## 1. Introduction

Fiber-reinforced polymer (FRP) composites combine high specific strength and stiffness with corrosion resistance and design flexibility, supporting their use in aerospace, automotive, and rail structures [1,2,3,4]. Carbon fiber-reinforced polymers (CFRPs) provide high load capacity and mass savings in stiffness-critical applications [4], whereas glass fiber-reinforced polymers (GFRPs) offer lower cost, higher failure strain, and mature manufacturing routes [5]. Laminated FRPs nevertheless exhibit pronounced in-plane and through-thickness anisotropy [6]: continuous fibers carry most in-plane loads, whereas through-thickness load transfer depends mainly on the matrix and fiber–matrix interface [7]. Interlaminar stresses at free edges, ply drop-offs, geometric discontinuities, and impact sites can therefore initiate delamination [8], which may remain barely visible while reducing stiffness and residual load capacity through interactions among buckling, matrix cracking, and fiber fracture [9,10,11]. Enhancing interlaminar fracture resistance without compromising in-plane properties, mass, or manufacturability remains a central challenge [8,12]. Mode I and Mode II fracture toughnesses, measured by double cantilever beam (DCB) and end-notched flexure (ENF) testing according to ASTM D5528 and ASTM D7905, respectively [13,14], characterize resistance to opening- and shear-driven delamination. Both are required because service damage commonly combines these components [15,16,17].

Interlaminar toughening strategies include through-thickness reinforcement, ductile interlayers, and resin or interface modification [18,19]. Z-pinning, stitching, and three-dimensional weaving dissipate energy through bridging, pull-out, and friction [20,21], but may distort fibers or create resin-rich regions that impair in-plane performance [12]. Thermoplastic and nonwoven interlayers promote plastic deformation, crack deflection, and fiber bridging [22,23], although their effectiveness depends on thickness, areal density, wetting, and processing [24,25]. At smaller length scales, nanofillers can similarly promote matrix deformation, crack deflection, and particle debonding without perforating the laminate [26]. Localizing them within an interlayer can preserve the in-plane fiber architecture [12]. Carbon nanotubes (CNTs) are attractive because their high aspect ratio and strength enable multiscale bridging networks at low contents [8].

CNT interlayers can be introduced by dispersing CNTs in resin, depositing them on the reinforcement, incorporating CNT films or veils, growing CNTs on fiber surfaces, or hybridizing them with electrospun nanofibers [27,28]. Reported mechanisms include crack deflection and branching, CNT bridging, interfacial debonding, pull-out or rupture, and matrix shear yielding [29,30]. Their effectiveness depends on CNT content, morphology, orientation, and fracture mode. CNT veils aligned parallel to the crack front promoted tortuous crack paths and carbon-fiber bridging, increasing Mode I propagation toughness by approximately 107% [31], whereas spatially controlled CNT architectures produced improvements exceeding 60% [32]. The effects of fracture mode can oppose one another: a noncovalently modified CNT film increased CFRP Mode I toughness by approximately 206% but reduced Mode II toughness by about 8% [33], whereas magnetically aligned through-thickness CNTs enhanced Mode II resistance, with 0.3 wt.% producing the highest response among the tested concentrations [34]. Thus, a Mode I gain does not necessarily imply a comparable Mode II response. More importantly, CNT content commonly produces a nonmonotonic shift in the dominant fracture features. At low content, few active nanotube–matrix interfaces limit bridging and frictional dissipation; at intermediate content, distributed pull-out, crack deflection, and matrix deformation become more extensive; and at excessive content, increased viscosity, impaired wetting, bundling, and agglomeration can create weak regions that favor defect-assisted crack growth. Increases followed by declines have been reported for spray-deposited CNT contents of 0.05–0.20 wt.% [35] and for dual-mode toughness with increasing CNT areal density [36]. The effective content must therefore be established for each combination of fiber system, resin, and processing route [37].

Carbon/glass hybrid laminates combine the high modulus and low density of carbon fibers with the lower cost, higher failure strain, and impact-energy absorption of glass fibers [38]. Their interlaminar fracture behavior cannot, however, be inferred directly from constituent properties because tow orientation, stiffness mismatch, and the precracked interface affect crack initiation and migration [17,39]. Relatively few studies have systematically characterized both DCB and ENF behavior in hybrid composites [40], and research on CNT-toughened hybrid systems has largely emphasized orientation effects or damage sensing [41]. It therefore remains unclear whether concentration-dependent CNT toughening, established mainly for unidirectional CFRP, transfers to CFRP, GFRP, and carbon/glass hybrids. Differences in fiber surface chemistry, interlaminar resin thickness, and crack-path topology may prevent direct transfer [42], while Mode I and Mode II studies often use different CNT forms, contents, processing routes, and laminate architectures. A controlled comparison across multiple fiber systems, both fracture modes, and a common MWCNT concentration range is therefore required [39] to separate concentration effects from material- and mode-dependent crack paths.

Accordingly, this study compares Mode I propagation and Mode II initiation toughness in CFRP, GFRP, and two carbon/glass hybrid laminate orientations containing 0.1, 0.3, or 0.5 wt.% MWCNT, together with unmodified and neat-epoxy-interlayer baselines. All groups were manufactured using the same DMF-assisted ultrasonication and hot-pressing route, tested by DCB and ENF methods, and examined by scanning electron microscopy (SEM). The study evaluates whether increasing MWCNT content shifts the dominant fracture features from limited nanotube participation, through distributed crack deflection, pull-out, and matrix deformation, to increasingly bundle- or agglomerate-related defect-assisted fracture, and how fiber architecture and loading mode modify this transition.

## 2. Materials and Methods

### 2.1. Materials

Four laminate configurations were investigated: carbon fiber-reinforced polymer (CFRP), glass fiber-reinforced polymer (GFRP), and two carbon/glass hybrid systems (HCRP and HGRP). All laminates were manufactured from commercial epoxy-based plain-weave prepregs with a nominal uncured ply thickness of 0.25 mm. All prepregs were supplied by Yixing Ruibang Composite Materials Co., Ltd. (Yixing, Jiangsu, China). The CFRP and GFRP prepregs comprised T300 carbon-fiber plain-weave fabric (200 g/m^2^) and E-glass plain-weave fabric (300 g/m^2^), respectively. The hybrid prepreg consisted of a co-woven carbon/glass plain-weave fabric with carbon and glass tows in the warp and weft directions, respectively. For HCRP, the carbon tows were aligned with the longitudinal specimen axis. The same hybrid prepreg was rotated by 90° for HGRP, thereby aligning the glass tows longitudinally. Table 1 summarizes the basic properties of the T300 carbon and E-glass fibers.

Carboxyl-functionalized multi-walled carbon nanotubes (MWCNTs) were supplied by Shenzhen Suiheng Technology Co., Ltd. (Shenzhen, China). The MWCNTs had an inner diameter of 3–5 nm, an outer diameter of 8–15 nm, a length of 5–15 μm, and a purity greater than 98%, and were used as the interlaminar modifier. The two-part epoxy system comprised an epoxy resin (Part A) and a curing agent (Part B) mixed at a 2:1 mass ratio. Reagent-grade N,N-dimethylformamide (DMF; purity ≥ 99.5%) was used as the dispersion solvent, and a 20-μm-thick polyimide film was used as the starter film.

### 2.2. Preparation of MWCNT-Modified Epoxy Systems

**Figure 1:**
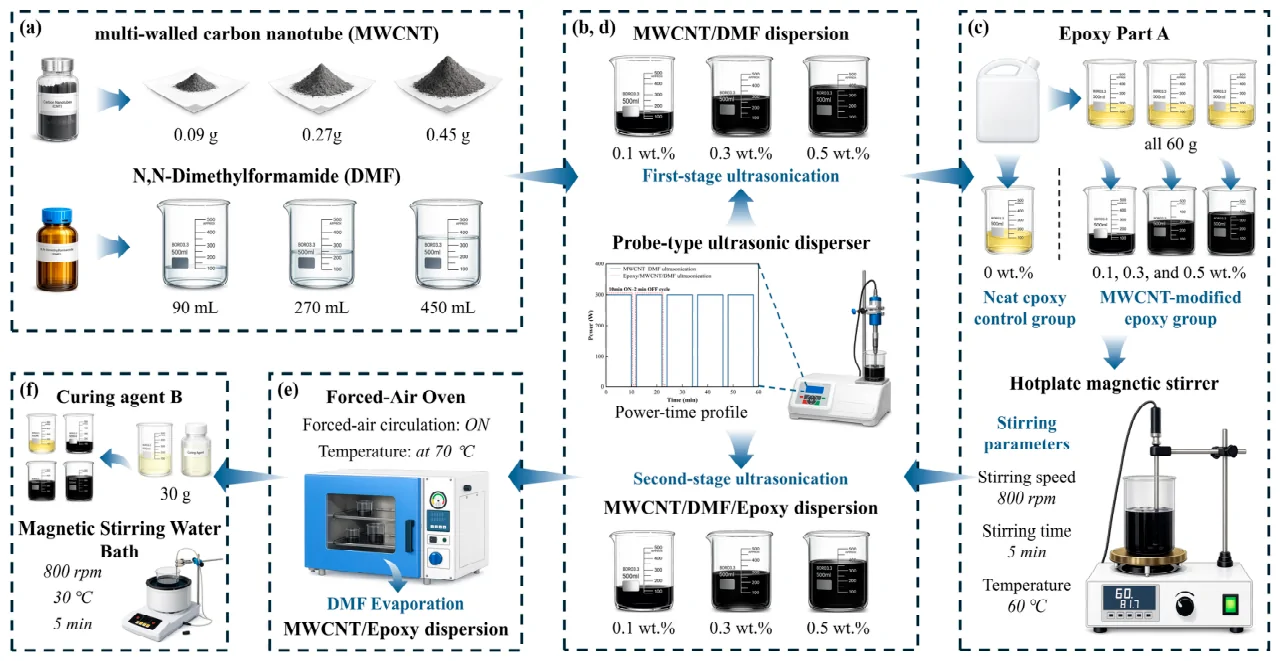
Preparation of the MWCNT-modified epoxy systems: (**a**) measurement of the MWCNTs and DMF; (**b**) first-stage ultrasonication of MWCNTs in DMF; (**c**) addition of epoxy resin (Part A) and heated magnetic stirring; (**d**) second-stage ultrasonication; (**e**) DMF evaporation; and (**f**) addition and mixing of the curing agent (Part B).

Three MWCNT-modified epoxy systems containing nominal MWCNT concentrations of 0.1, 0.3, or 0.5 wt.% were prepared using a DMF-assisted ultrasonication procedure. A neat-epoxy system containing 0 wt.% MWCNT served as the interlayer control. The preparation procedure is summarized in Figure 1.

For a total resin mass of 90 g, MWCNT masses of 0.09, 0.27, and 0.45 g were dispersed in 90, 270, and 450 mL of DMF, respectively, thereby maintaining a constant MWCNT-mass-to-DMF-volume ratio. Each MWCNT/DMF suspension was ultrasonicated at 300 W for six cycles, each comprising 10 min of sonication followed by a 2-min rest interval. The rest intervals limited excessive heating during ultrasonication.

After the first ultrasonication stage, 60 g of Part A was added directly to each suspension. The resulting MWCNT/DMF/epoxy mixtures were magnetically stirred at 800 rpm and 60 °C for 5 min and then subjected to a second ultrasonication stage comprising two 10-min cycles at 300 W. The dispersion state was inspected visually during preparation, and mixtures without obvious large CNT clusters were accepted for subsequent processing.

The MWCNT-containing mixtures were then placed in a forced-air oven at 70 °C under continuous air circulation for 3–4 days, until DMF evaporation was judged complete by visual inspection. Subsequently, 30 g of Part B was added to each MWCNT/epoxy mixture. The neat-epoxy control was prepared without MWCNTs or DMF: 60 g of Part A was magnetically stirred at 800 rpm and 60 °C for 5 min, after which 30 g of Part B was added. Before laminate fabrication, all four resin systems were magnetically stirred at 800 rpm and 30 °C for 5 min in a temperature-controlled water bath.

### 2.3. Laminate Fabrication and Specimen Preparation

**Figure 2:**
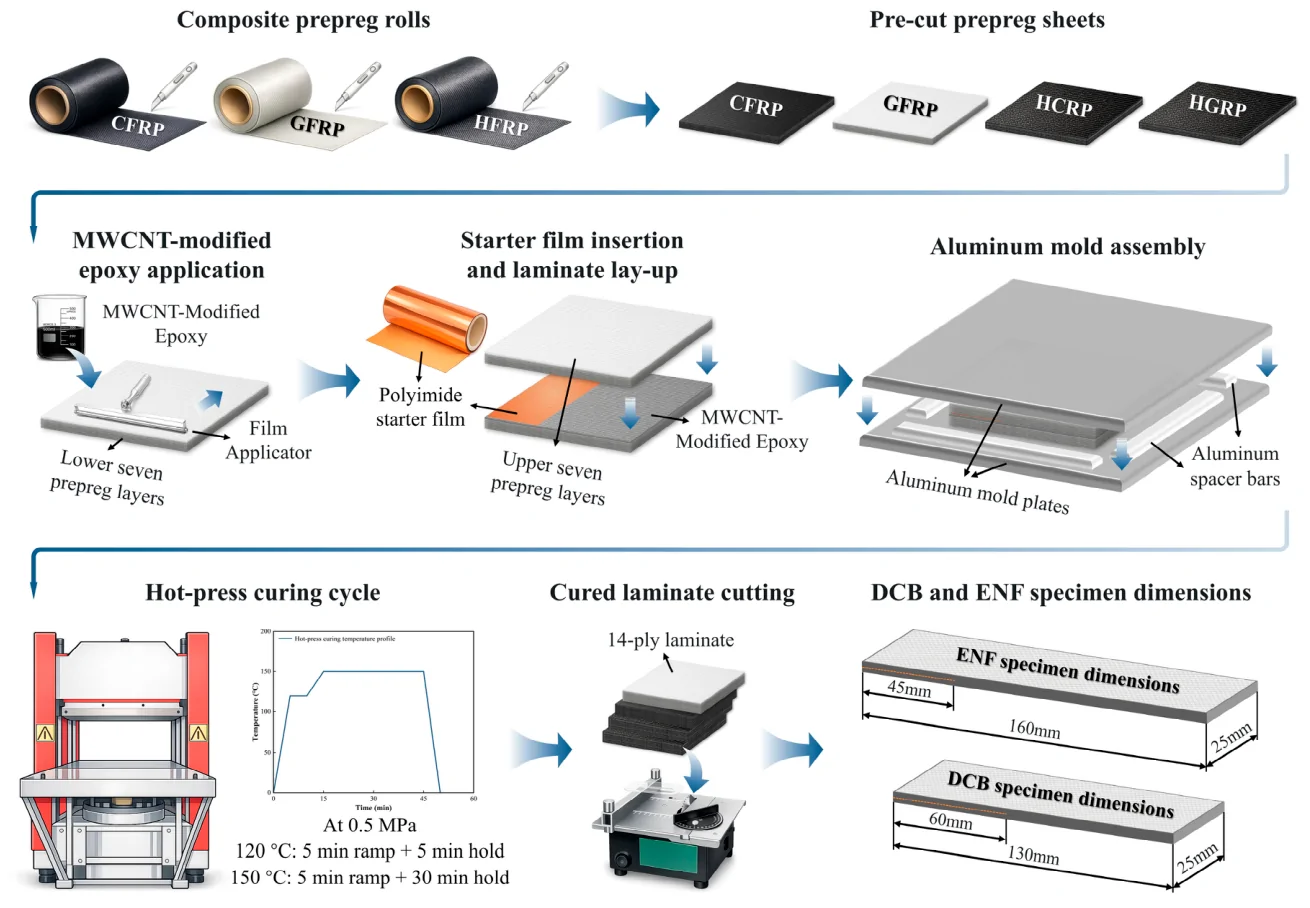
Laminate fabrication and specimen preparation for the four material configurations, including prepreg cutting, symmetric 14-ply manual lay-up, midplane application of the neat or MWCNT-modified epoxy interlayer using a film applicator where applicable, starter-film insertion, hot-press consolidation, and cutting of the DCB and ENF specimens.

The plain-weave prepregs were cut into rectangular sheets and manually stacked into symmetric 14-ply laminates, as illustrated in Figure 2. For the MWCNT-modified and neat-epoxy control laminates, a continuous layer of the corresponding formulation was spread over the upper surface of the seventh ply using a film applicator. A 20-μm-thick polyimide starter film was inserted at the midplane between the seventh and eighth plies. The insert lengths were 60 mm for the double cantilever beam (DCB) specimens and 45 mm for the end-notched flexure (ENF) specimens. The remaining seven plies were then added, and the lay-up was placed between aluminum mold plates fitted with spacer bars to control laminate thickness.

The laminates were consolidated in a hot press under a constant pressure of 0.5 MPa. The mold was preheated to 90 °C, heated to 120 °C over 5 min, and held at 120 °C for 5 min. The temperature was then increased to 150 °C over a further 5 min and held at 150 °C for 30 min. After curing, the laminates were allowed to cool naturally to room temperature.

The resulting 14-ply laminates were approximately 3.5 mm thick. A precision table saw was used to cut DCB specimens measuring 130 mm × 25 mm × 3.5 mm and ENF specimens measuring 160 mm × 25 mm × 3.5 mm.

Five interlayer conditions were prepared for each material system: an unmodified laminate (ORI) without an added resin interlayer; a control laminate (CON) containing a neat-epoxy interlayer without MWCNTs; and laminates containing 0.1, 0.3, or 0.5 wt.% MWCNT in the interlayer. CON was included to distinguish the effect of the added epoxy layer from that of MWCNT addition. For each combination of laminate system, interlayer condition, and fracture test, one laminate panel was manufactured and cut into three test specimens, yielding 40 panels and 120 specimens.

### 2.4. Mode I Interlaminar Fracture Testing

**Figure 3:**
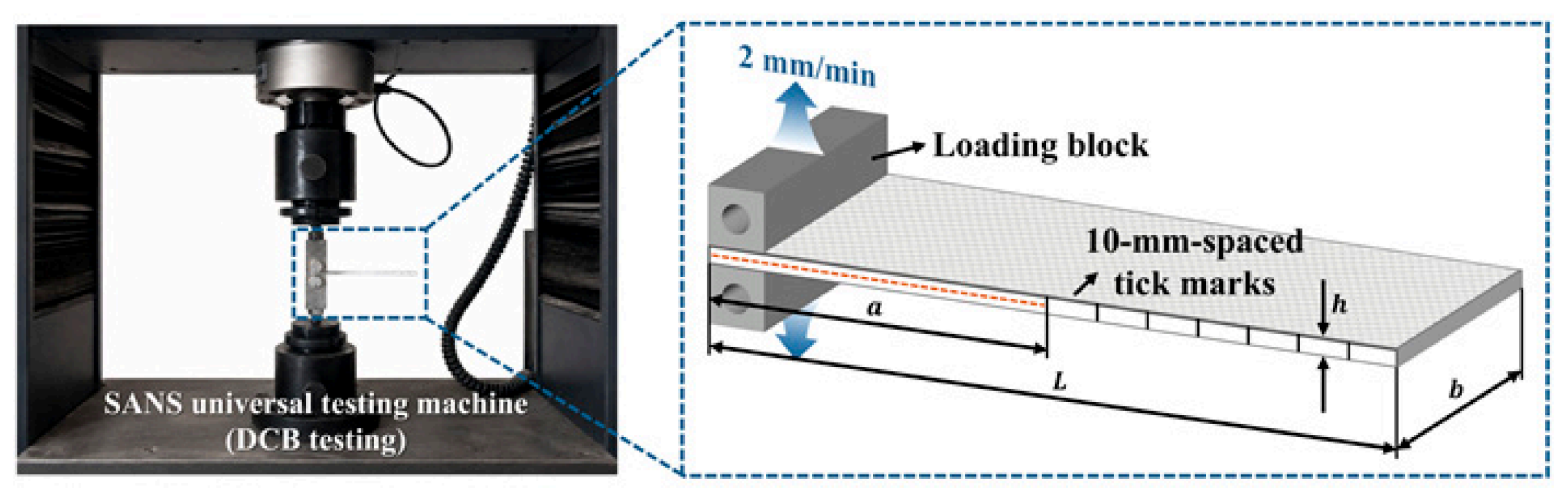
Mode I DCB test setup and specimen geometry, showing the loading blocks, opening direction, crosshead rate (2 mm/min), starter-film length *a*, 10-mm-spaced tick marks, specimen length *L*, thickness *h*, and width *b*.

Mode I interlaminar fracture resistance was measured by DCB testing according to ASTM D5528 (Figure 3). Metallic loading blocks were adhesively bonded to the upper and lower surfaces at the starter-film end of each specimen. One lateral edge was painted white and marked at 10-mm intervals to improve crack-tip visibility and facilitate crack-length measurement.

The tests were performed on a SANS universal testing machine at a crosshead rate of 2 mm/min. Load and displacement were recorded continuously, and crack propagation was imaged at 1 frame/s. The instantaneous delamination length *a′*, defined as the distance from the loading line to the crack tip, was measured frame by frame using ImageJ (version 1.54g) and synchronized with the corresponding load and displacement data.

The Mode I strain energy release rate *G_Ic_* was calculated using modified beam theory. Specimen compliance *C* was defined as

(1) $$C=\frac{\delta }{P}$$

where *δ* is the load-point displacement, and *P* is the applied load. The cube root of compliance, *C*^1/3^, was linearly regressed against *a′*. The magnitude of the x-intercept was taken as the crack-length correction |Δ| to account for beam-arm rotation at the delamination front. The Mode I strain energy release rate was then calculated from

(2) $$G_{\mathrm{Ic}}=\frac{3P\delta }{2b\left(a^{\prime }+\left|\Delta \right|\right)}$$

where *b* is the specimen width. Three DCB specimens were tested for each laminate system and interlayer condition. The calculated *G_Ic_* values were plotted against *a′* to obtain resistance curves. For each specimen, the steady-state value, denoted *G_s_*, was determined from the stable plateau region of its resistance curve. The three specimen values were subsequently summarized as the mean and standard deviation for each configuration.

### 2.5. Mode II Interlaminar Fracture Testing

**Figure 4:**
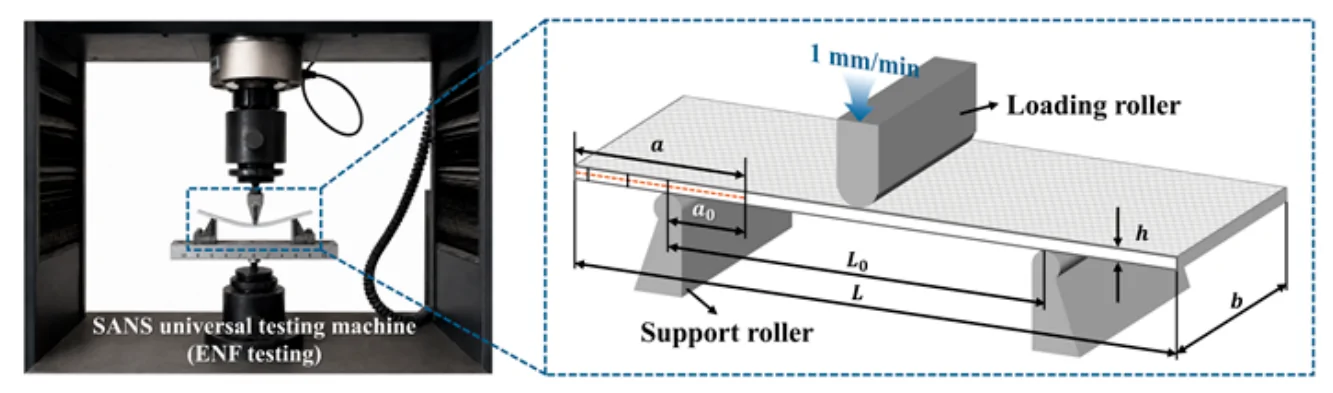
Mode II ENF test setup and specimen configuration, showing the central loading roller, support rollers, loading direction, crosshead rate (1 mm/min), starter-film length *a*, effective crack length *a*_0_, support span *L*_0_, specimen length *L*, thickness *h*, and width *b*.

Mode II interlaminar fracture resistance was measured by ENF testing according to ASTM D7905 (Figure 4). Each specimen was supported by two cylindrical rollers with a support span *L*_0_ of 100 mm and loaded at midspan by a central roller. The tests were performed on a SANS universal testing machine at a crosshead rate of 1 mm/min.

For each ENF specimen, compliance-calibration loading was conducted at effective crack lengths *a*_0_ of 20, 30, and 40 mm. At each calibration position, the specimen was loaded within the linear-elastic range and then unloaded. The fracture test was subsequently conducted at *a*_0_ = 30 mm, with loading continued until the onset of unstable delamination.

The compliance at each crack length was obtained as the inverse slope of the initial linear portion of the corresponding load–displacement record. The initial linear portion of the 30-mm fracture-test record provided the third compliance value. Thus, three specimen-specific compliance values, corresponding to *a*_0_ = 20, 30, and 40 mm, were obtained for each ENF specimen and linearly regressed against the cube of the corresponding crack length:

(3) $$C=A+ma_{0}^{3}$$

where *A* is the fitted intercept, and *m* is the specimen-specific compliance-calibration coefficient, corresponding to the slope of the *C*-$a_{0}^{3}$ relationship.

For the 30-mm fracture tests, the maximum load *P_max_*, associated with the onset of unstable delamination, was recorded. The Mode II initiation toughness *G_IIc_* was calculated as

(4) $$G_{\mathrm{IIc}}=\frac{3mP_{\max}^{2}a_{0}^{2}}{2b}$$

where *b* is the specimen width, and *a*_0_ = 30 mm. Three ENF specimens were tested for each material system and interlayer condition, and the resulting values were summarized as the mean and standard deviation.

### 2.6. Fractographic Analysis

Scanning electron microscopy (SEM) was used to examine fracture-surface features associated with the Mode I and Mode II responses. Before incorporation into the epoxy, the as-received MWCNT powder was mounted on conductive adhesive tape and sputter-coated with gold for 60 s.

Two complementary sets of composite fracture surfaces were examined. For the concentration-dependent comparison, GFRP specimens tested under Mode I and HCRP specimens tested under Mode II were examined for all five interlayer conditions. For the cross-material Mode II comparison, ENF fracture surfaces of CFRP, GFRP, HCRP, and HGRP specimens containing 0.3 or 0.5 wt.% MWCNT were examined.

For each selected specimen, an approximately 8 mm × 8 mm section was cut from a region located 10–30 mm beyond the polyimide starter film along the crack-growth direction. The sections were ultrasonically cleaned in ethanol, dried at room temperature, and sputter-coated with gold for 60 s to reduce charging of the nonconductive epoxy-rich matrix. SEM observations were conducted at an accelerating voltage of 5.00 kV and a working distance of 11.0 mm. For the matched fracture-surface comparisons, low- and high-magnification micrographs were acquired at ×2000 and ×10,000, respectively, under consistent imaging conditions to document the overall fracture morphology and local features.

### 2.7. Statistical Analysis

For each configuration, three specimen-level toughness values were obtained and reported as the mean ± standard deviation (SD). Because the three specimens within each configuration were cut from a single manufactured panel, the specimen was treated as the unit of analysis; statistical inference therefore represents within-panel specimen-level variability.

For each MWCNT-modified group, the percentage change in fracture toughness relative to the corresponding CON group was calculated as

(5) $$\Delta G_{i}\left(\%\right)=\frac{\bar{G_{i}}-{\bar{G}}_{\mathrm{CON}}}{{\bar{G}}_{\mathrm{CON}}}\times 100$$

where $\bar{G_{i}}$ and ${\bar{G}}_{\mathrm{CON}}$ are the mean toughness values of the MWCNT-modified group and the corresponding CON group, respectively. *G_s_* was used for Mode I, and *G_IIc_* was used for Mode II.

The effect of MWCNT concentration was evaluated separately for each laminate system and fracture mode using Welch’s one-way analysis of variance across CON and the 0.1, 0.3, and 0.5 wt.% MWCNT groups. Games–Howell post hoc comparisons were performed between the 0.3 wt.% group and CON, 0.1 wt.%, and 0.5 wt.% groups. Pairwise comparisons were interpreted as confirmatory only when the corresponding omnibus test reached statistical significance. The significance level was set at *α* = 0.05, and exact *p*-values were reported. Statistical calculations were performed using custom scripts in Python 3.13.5 with SciPy 1.17.0.

## 3. Results

### 3.1. Mode I Interlaminar Fracture Behavior

#### 3.1.1. DCB Load–Displacement Response

**Figure 5:**
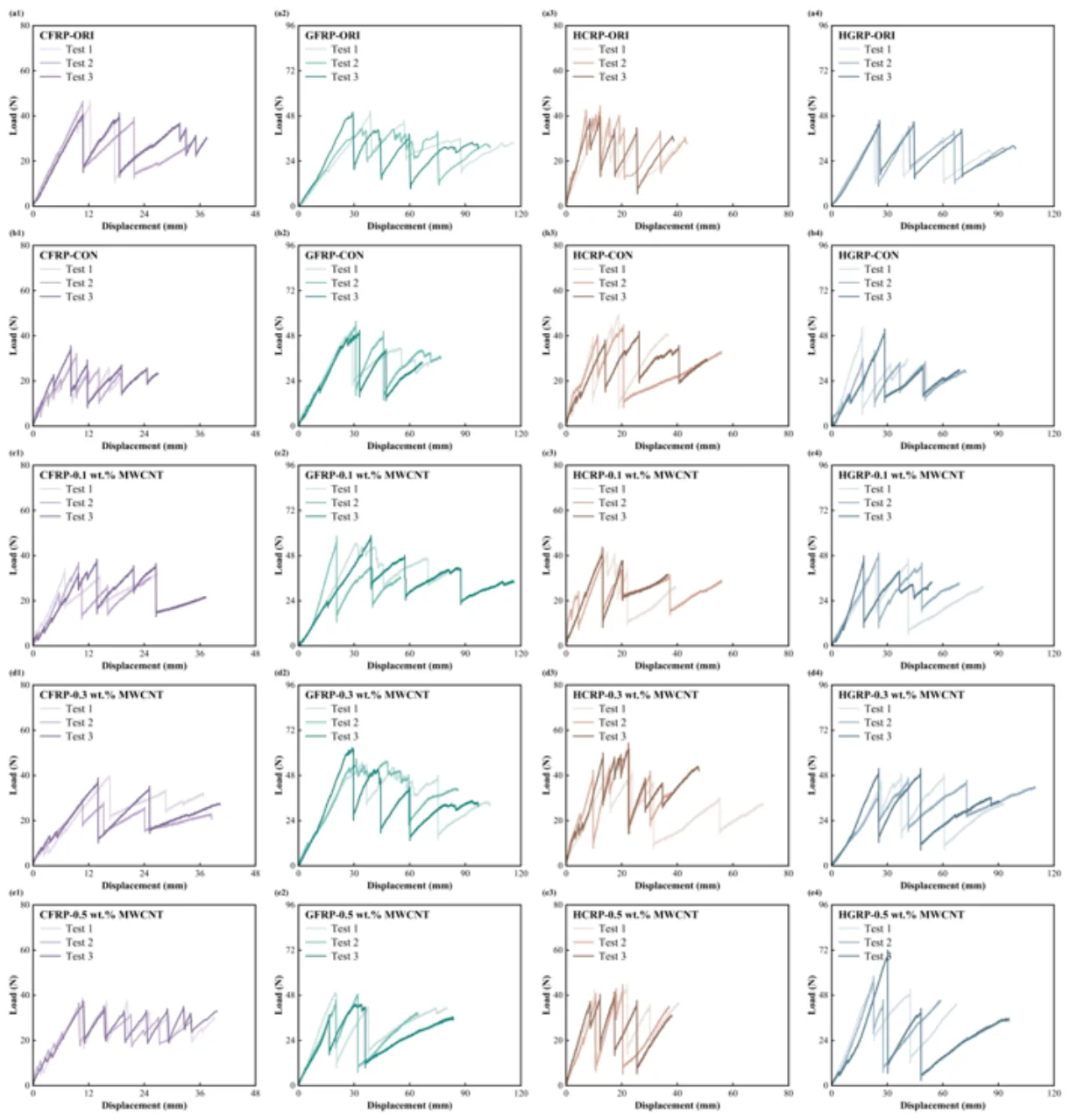
DCB load–displacement curves for the four material systems and five interlayer conditions (three specimens per configuration). Rows: (**a1**–**a4**) ORI, (**b1**–**b4**) CON, and interlayers containing (**c1**–**c4**) 0.1 wt.%, (**d1**–**d4**) 0.3 wt.%, and (**e1**–**e4**) 0.5 wt.% MWCNT. Columns: (**a1**–**e1**) CFRP, (**a2**–**e2**) GFRP, (**a3**–**e3**) HCRP, and (**a4**–**e4**) HGRP.

Figure 5 presents the DCB load–displacement curves for the four material systems and five interlayer conditions. All responses were initially linear, followed by crack initiation and progressive propagation with a gradual reduction in load-carrying capacity. Pronounced serrations occurred after initiation in all configurations, indicating repeated stick–slip crack growth during delamination.

The CFRP specimens (Figure 5(a1–e1)) exhibited a substantially smaller displacement range than the GFRP (Figure 5(a2–e2)) and HGRP (Figure 5(a4–e4)) specimens. The HCRP specimens (Figure 5(a3–e3)), in which the carbon tows were aligned longitudinally, showed displacement ranges and overall load responses similar to those of CFRP. In contrast, GFRP and HGRP exhibited larger displacement ranges and generally higher peak loads. Across the four material systems, the peak load generally increased from the ORI and CON baselines to the 0.3 wt.% condition and then decreased at 0.5 wt.%, although the latter difference was not distinct in every configuration. The three curves within each configuration were closely grouped, indicating good within-panel repeatability.

#### 3.1.2. Mode I Resistance Curves

**Figure 6:**
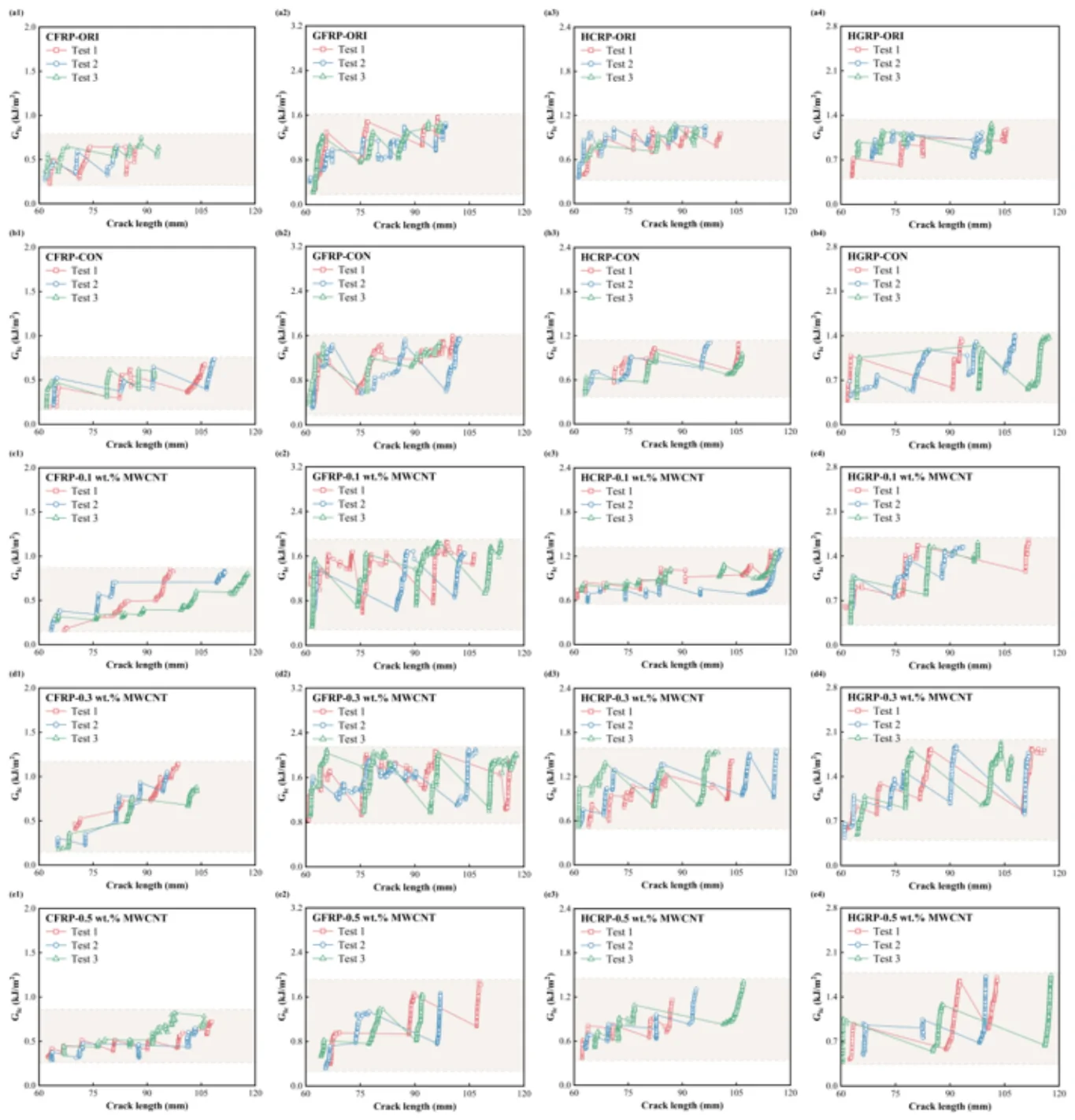
Mode I resistance curves for the four laminate systems and five interlayer conditions, plotted as *G_Ic_* against crack length for three DCB specimens per configuration. Rows: (**a1**–**a4**) ORI, (**b1**–**b4**) CON, (**c1**–**c4**) 0.1 wt.% MWCNT, (**d1**–**d4**) 0.3 wt.% MWCNT, and (**e1**–**e4**) 0.5 wt.% MWCNT. Columns correspond to CFRP, GFRP, HCRP, and HGRP, respectively. Red squares, blue circles, and green triangles denote specimens 1, 2, and 3. Shaded regions bounded by dashed lines indicate the *G_Ic_* ranges.

Mode I resistance curves generally increased during the early stages of propagation and approached plateaus at longer crack lengths (Figure 6), indicating the development of steady-state crack-growth resistance. Local fluctuations were superimposed on both the rising and plateau regions and corresponded to the stick–slip responses in Figure 5. Despite these fluctuations, the three specimens within most configurations followed comparable ranges and concentration-dependent trends.

Within each material system, ORI and CON exhibited similar overall resistance levels. Among the MWCNT-modified groups, the plateau levels generally increased from 0.1 to 0.3 wt.% and then decreased at 0.5 wt.%, with the 0.3 wt.% condition exhibiting the highest plateau levels across the four systems. In some configurations, the 0.5 wt.% curves approached the baseline range. Overall, the resistance curves showed a consistent nonmonotonic concentration-dependent trend despite differences in absolute toughness among the material systems.

#### 3.1.3. Effect of MWCNT Concentration on Steady-State Mode I Toughness

**Figure 7:**
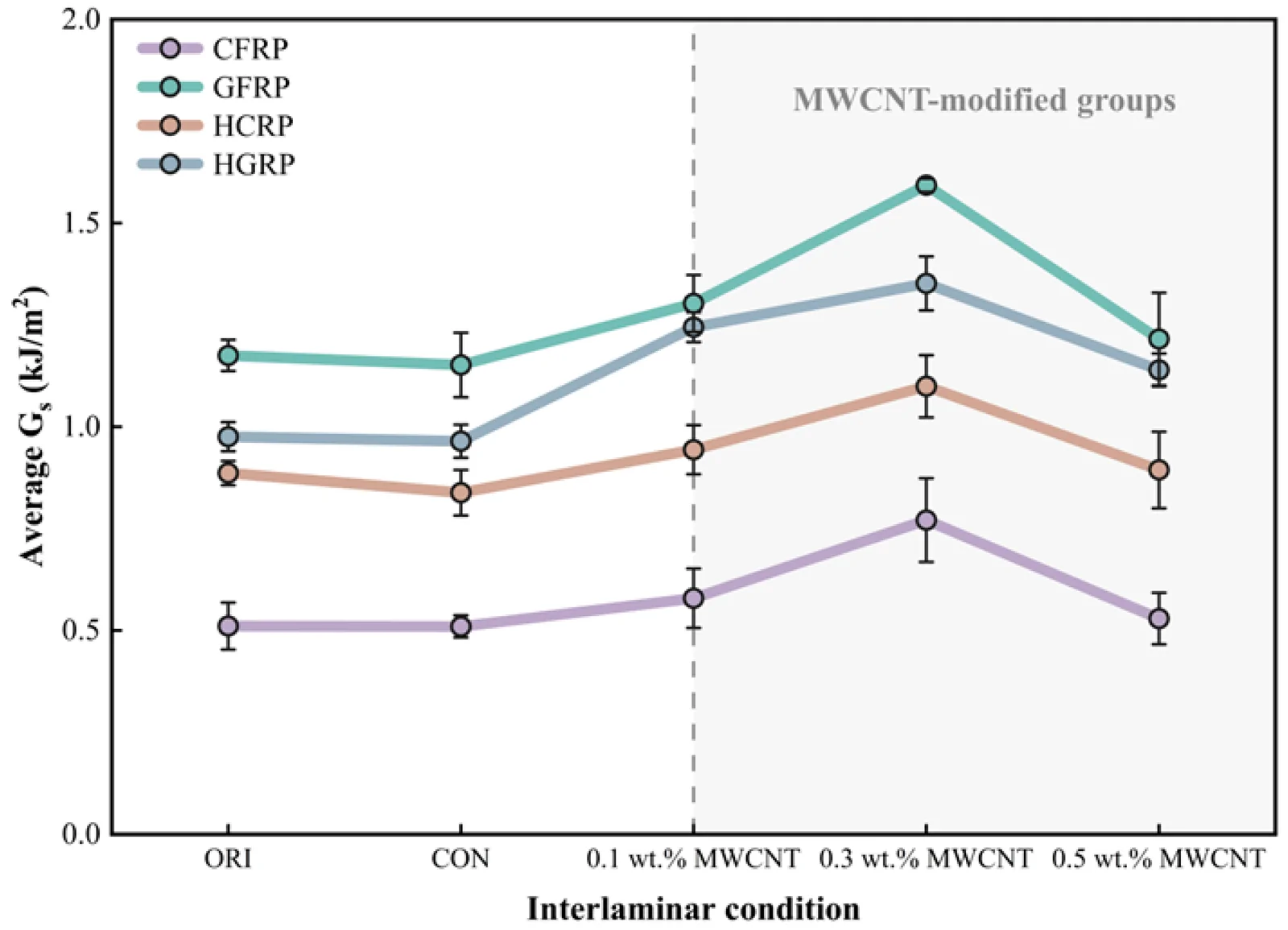
Mean steady-state Mode I interlaminar fracture toughness (*G_s_*) of CFRP, GFRP, HCRP, and HGRP under the ORI, CON, and 0.1, 0.3, and 0.5 wt.% MWCNT conditions. Symbols and error bars represent the mean and standard deviation, respectively, for three DCB specimens. The dashed line separates the baseline and MWCNT-modified groups.

Figure 7 and Table 2 summarize the mean steady-state Mode I toughness (*G_s_*) for the four material systems and five interlayer conditions. GFRP exhibited the highest mean *G_s_* under every condition, followed by HGRP, HCRP, and CFRP; this ordering was maintained in both the baseline and MWCNT-modified groups. Within each material system, ORI and CON had comparable mean *G_s_* values. Their respective means were 0.511 and 0.510 kJ/m^2^ for CFRP, 1.175 and 1.152 kJ/m^2^ for GFRP, 0.886 and 0.838 kJ/m^2^ for HCRP, and 0.976 and 0.965 kJ/m^2^ for HGRP, indicating little change from the neat-epoxy interlayer alone.

All four material systems showed a nonmonotonic response to MWCNT content. Relative to CON, 0.1 wt.% MWCNT increased *G_s_* by 13.6%, 13.1%, 12.6%, and 29.0% for CFRP, GFRP, HCRP, and HGRP, respectively. The highest mean values were obtained at 0.3 wt.%: 0.771 ± 0.103, 1.593 ± 0.016, 1.099 ± 0.077, and 1.352 ± 0.066 kJ/m^2^ in the same material order. These values corresponded to increases of 51.2%, 38.3%, 31.2%, and 40.1% relative to CON. GFRP therefore retained the highest absolute Mode I toughness, whereas CFRP exhibited the largest relative increase.

At 0.5 wt.%, the mean *G_s_* values decreased to 0.529, 1.215, 0.894, and 1.139 kJ/m^2^ for CFRP, GFRP, HCRP, and HGRP, respectively. These values remained 3.8%, 5.5%, 6.7%, and 18.1% above CON. Thus, the decline from 0.3 wt.% represented a reduction in toughening benefit, although HGRP retained a comparatively larger improvement over its baseline. Despite differences in absolute toughness and enhancement magnitude, all four systems exhibited the same concentration-dependent Mode I trend.

### 3.2. Mode II Interlaminar Fracture Behavior

#### 3.2.1. ENF Load–Displacement Response

**Figure 8:**
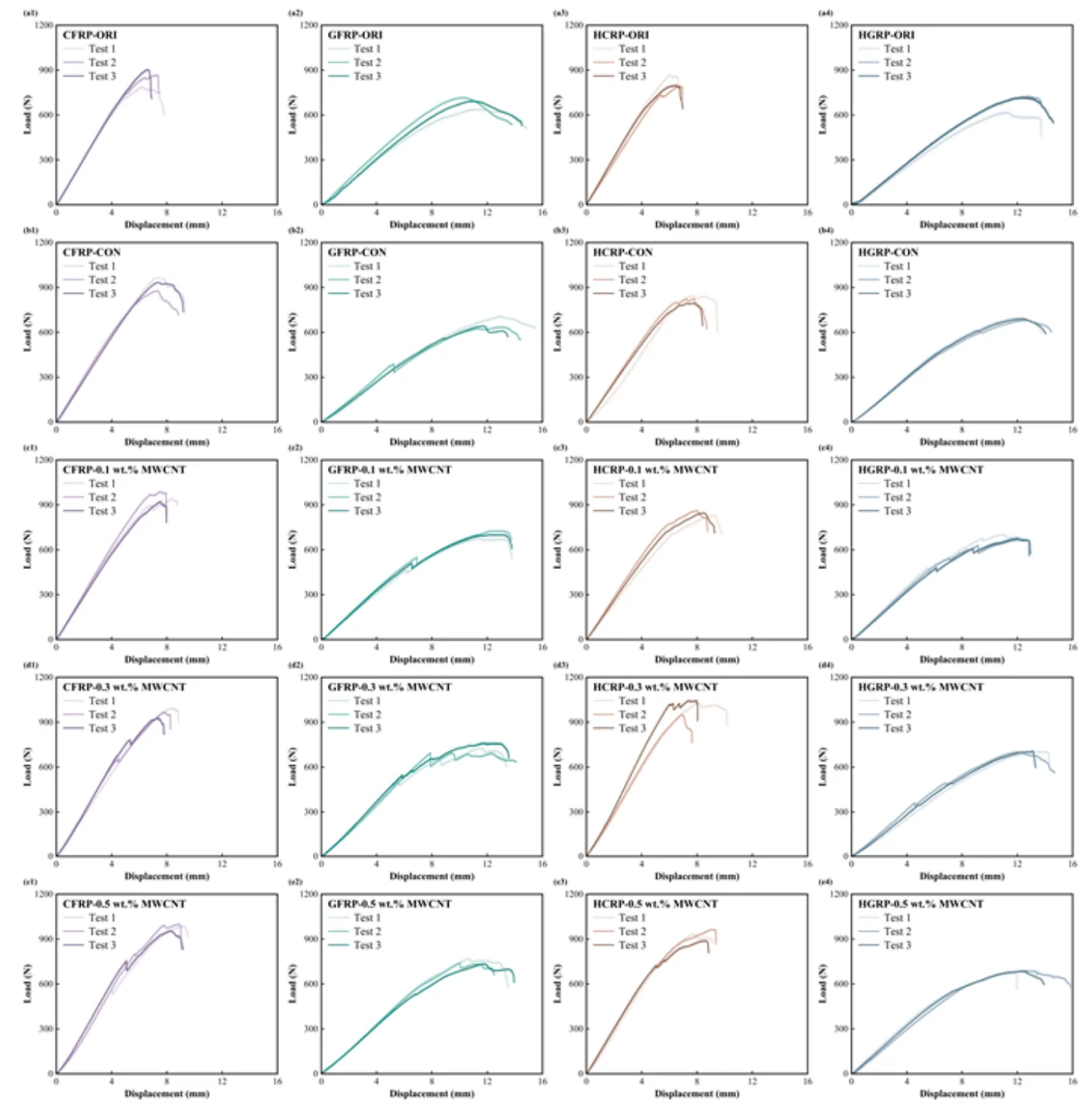
ENF load–displacement curves for the four material systems and five interlayer conditions (three specimens per configuration). Rows: (**a1**–**a4**) ORI, (**b1**–**b4**) CON, and interlayers containing (**c1**–**c4**) 0.1 wt.%, (**d1**–**d4**) 0.3 wt.%, and (**e1**–**e4**) 0.5 wt.% MWCNT. Columns: (**a1**–**e1**) CFRP, (**a2**–**e2**) GFRP, (**a3**–**e3**) HCRP, and (**a4**–**e4**) HGRP.

Figure 8 presents the ENF load–displacement curves for the four material systems and five interlayer conditions, with three specimens per configuration. Each curve comprised an initially quasi-linear region, a distinct peak load, and a subsequent load drop associated with the onset of Mode II crack growth. The quasi-linear region corresponded to elastic bending before crack advance, whereas the peak marked the onset of delamination. Unlike the stable-propagation DCB analysis, each ENF curve therefore provided a single initiation value.

The overall curve shape was similar across material systems and interlayer conditions. The four material systems exhibited broadly comparable displacement ranges, typically 0–16 mm, and peak loads of approximately 600–900 N. Intermaterial differences were less pronounced than those observed in the DCB tests. Peak load generally increased from ORI and CON to the 0.3 wt.% condition and then decreased at 0.5 wt.% in most systems, consistent with the Mode I trend. The three curves within each configuration overlapped closely, indicating good within-panel repeatability.

#### 3.2.2. Effect of MWCNT Concentration on Mode II Toughness

**Figure 9:**
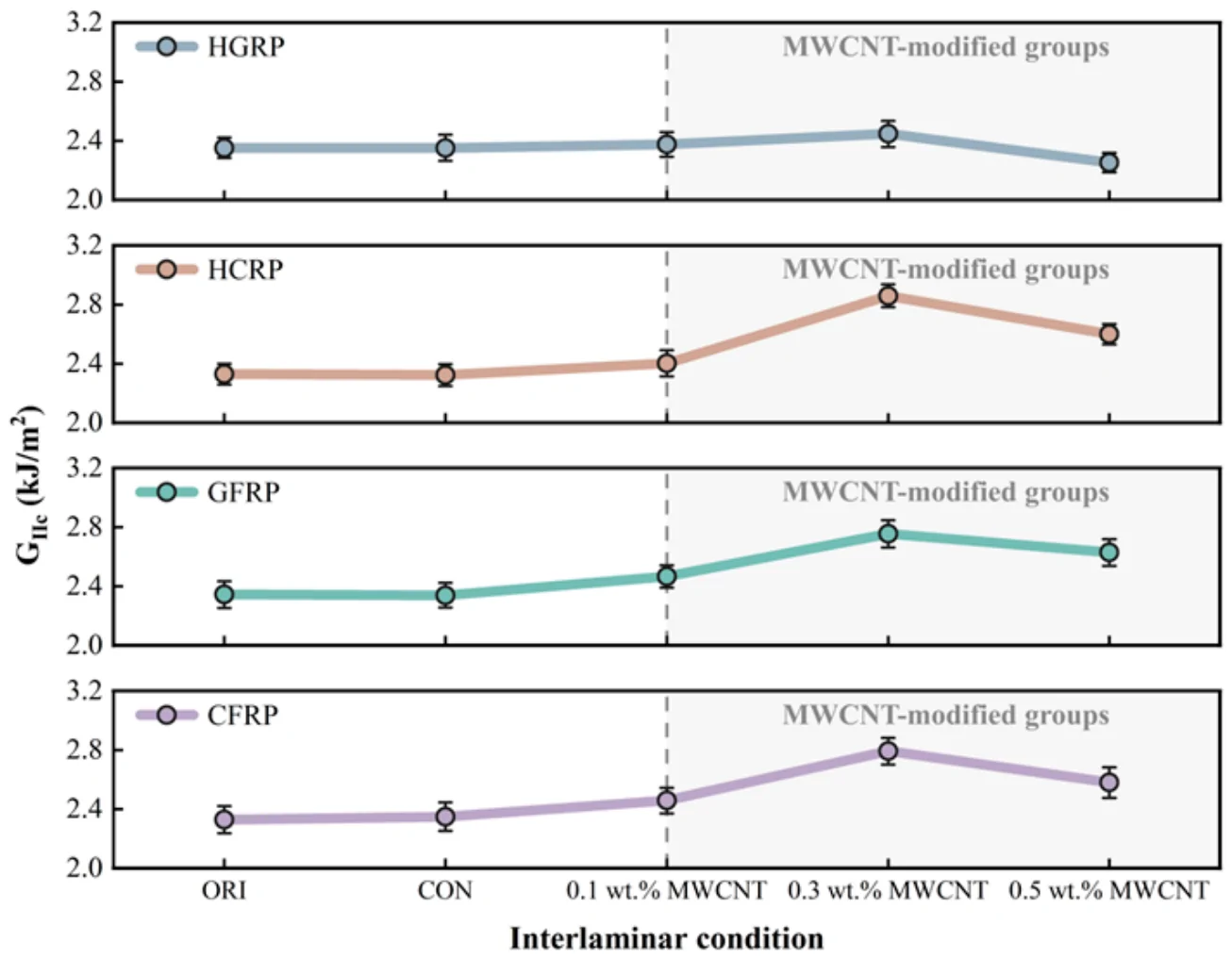
Mean Mode II interlaminar fracture toughness (*G_IIc_*) of CFRP, GFRP, HCRP, and HGRP under the ORI, CON, and 0.1, 0.3, and 0.5 wt.% MWCNT conditions. Symbols and error bars represent the mean and standard deviation, respectively, for three ENF specimens. The dashed line separates the baseline and MWCNT-modified groups.

Figure 9 and Table 3 summarize the mean Mode II initiation toughness (*G_IIc_*) of the four material systems across the five interlayer conditions. As in Mode I, ORI and CON exhibited comparable *G_IIc_* values within each material system. Their respective means were 2.331 and 2.349 kJ/m^2^ for CFRP, 2.345 and 2.339 kJ/m^2^ for GFRP, 2.330 and 2.323 kJ/m^2^ for HCRP, and 2.353 and 2.353 kJ/m^2^ for HGRP, again indicating little contribution from the neat-epoxy interlayer alone.

At 0.1 wt.%, the changes relative to CON were modest, ranging from 1.0% for HGRP to 5.4% for GFRP. The 0.3 wt.% condition gave the highest mean *G_IIc_* in every system: 2.792 ± 0.090, 2.755 ± 0.093, 2.859 ± 0.078, and 2.447 ± 0.088 kJ/m^2^ for CFRP, GFRP, HCRP, and HGRP, respectively. These values represented increases of 18.9%, 17.8%, 23.1%, and 4.0% relative to CON. HCRP exhibited both the highest absolute value and the largest improvement, whereas HGRP remained weakly responsive.

At 0.5 wt.%, the mean *G_IIc_* decreased to 2.581, 2.629, 2.600, and 2.254 kJ/m^2^ in the same material order. CFRP, GFRP, and HCRP remained 9.9%, 12.4%, and 11.9% above CON, whereas HGRP fell 4.2% below CON. Thus, the decline from 0.3 to 0.5 wt.% was common, but the residual response was material-dependent. This modal dependence was most evident for HGRP, for which toughness increased by 40.1% under Mode I but only by 4.0% under Mode II at 0.3 wt.%.

**Table 3:** Mode II interlaminar fracture toughness (mean ± SD, n = 3) and changes relative to CON for the four laminate systems.

Laminate System	Interlaminar Condition	*G_IIc_* (kJ/m^2^)	Change Relative to CON
		Mean ± SD	(%)
CFRP	ORI	2.331 ± 0.092	—
	CON	2.349 ± 0.096	—
	0.1 wt.% MWCNT	2.459 ± 0.086	+4.7
	0.3 wt.% MWCNT	2.792 ± 0.090	+18.9
	0.5 wt.% MWCNT	2.581 ± 0.104	+9.9
GFRP	ORI	2.345 ± 0.090	—
	CON	2.339 ± 0.084	—
	0.1 wt.% MWCNT	2.467 ± 0.076	+5.4
	0.3 wt.% MWCNT	2.755 ± 0.093	+17.8
	0.5 wt.% MWCNT	2.629 ± 0.091	+12.4
HCRP	ORI	2.330 ± 0.070	—
	CON	2.323 ± 0.074	—
	0.1 wt.% MWCNT	2.403 ± 0.089	+3.4
	0.3 wt.% MWCNT	2.859 ± 0.078	+23.1
	0.5 wt.% MWCNT	2.600 ± 0.069	+11.9
HGRP	ORI	2.353 ± 0.068	—
	CON	2.353 ± 0.088	—
	0.1 wt.% MWCNT	2.376 ± 0.082	+1.0
	0.3 wt.% MWCNT	2.447 ± 0.088	+4.0
	0.5 wt.% MWCNT	2.254 ± 0.066	−4.2

Percentage changes were calculated from the group means using Equation (5). Statistical comparisons are summarized in Table 4.

**Table 4:** Specimen-level statistical assessment of the ORI-CON baseline differences and MWCNT concentration effects on Mode I and Mode II interlaminar fracture toughness.

Fracture Mode	Laminate System	ORI vs. CON (*p*)	Welch ANOVA (*p*)	0.3 vs. CON (*p*)	0.3 vs. 0.1 wt.% (*p*)	0.3 vs. 0.5 wt.% (*p*)
Mode I	CFRP	0.9802	0.0832	0.1047	0.1876	0.1025
	GFRP	0.6816	**0.0034**	**0.0218**	**0.0393**	0.0678
	HCRP	0.2802	0.0520	0.0360 ^†^	0.1637	0.1381
	HGRP	0.7448	**0.0027**	**0.0068**	0.2346	**0.0427**
Mode II	CFRP	0.8262	**0.0203**	**0.0149**	**0.0328**	0.1736
	GFRP	0.9368	**0.0201**	**0.0159**	**0.0499** ^‡^	0.4371
	HCRP	0.9110	**0.0042**	**0.0035**	**0.0095**	**0.0427**
	HGRP	1.0000	0.1778	0.6041	0.7476	0.1288

ORI-CON comparisons used two-sided Welch’s *t*-tests. Concentration effects were evaluated by Welch’s one-way ANOVA across CON, 0.1, 0.3, and 0.5 wt.% MWCNT groups, followed by Games–Howell post hoc comparisons. Bold values indicate *p* < 0.05; post hoc values are highlighted only when the corresponding omnibus test was significant. ^†^ The corresponding omnibus test was not significant (*p* = 0.0520). ^‡^ Marginal comparison (*p* = 0.0499).

### 3.3. Statistical Assessment of Interlayer Effects

Table 4 distinguishes the descriptive ordering of the mean toughness values from statistically resolved differences among the interlayer conditions.

Welch’s *t*-tests detected no statistically significant difference between ORI and CON for any laminate system under either loading mode. The corresponding *p*-values ranged from 0.2802 to 0.9802 for Mode I and from 0.8262 to 1.0000 for Mode II. Thus, no specimen-level difference attributable solely to the added neat-epoxy interlayer was detected under the present processing and testing conditions.

Under Mode I loading, significant overall concentration effects were detected for GFRP, *F_W_*(3,3.557) = 38.921, *p* = 0.0034, and HGRP, *F_W_*(3,4.386) = 27.801, *p* = 0.0027, but not for CFRP, *F_W_*(3,3.925) = 4.826, *p* = 0.0832, or HCRP, *F_W_*(3,4.376) = 5.914, *p* = 0.0520. For GFRP, the 0.3 wt.% group was significantly higher than CON and 0.1 wt.%, whereas its difference from 0.5 wt.% was not significant. For HGRP, the 0.3 wt.% group was significantly higher than CON and 0.5 wt.%, but not 0.1 wt.%. Although 0.3 wt.% produced the highest mean *G_s_* in all four laminate systems, significant overall concentration effects were detected only for GFRP and HGRP.

Under Mode II loading, significant overall concentration effects were detected for CFRP, *F_W_*(3,4.435) = 9.855, *p* = 0.0203; GFRP, *F_W_*(3,4.431) = 9.912, *p* = 0.0201, and HCRP, *F_W_*(3,4.429) = 21.793, *p* = 0.0042, but not for HGRP, *F_W_*(3,4.411) = 2.602, *p* = 0.1778. The 0.3 wt.% CFRP group was significantly higher than CON and 0.1 wt.%, but not 0.5 wt.%. For GFRP, 0.3 wt.% was significantly higher than CON and marginally higher than 0.1 wt.%, whereas the difference from 0.5 wt.% was not significant. HCRP showed the clearest statistical separation: its 0.3 wt.% group was significantly higher than CON, 0.1 wt.%, and 0.5 wt.%. By contrast, none of the HGRP pairwise comparisons was significant.

Overall, 0.3 wt.% produced the highest mean toughness in all eight combinations, whereas significant overall concentration effects were detected in five. The descriptive maximum at 0.3 wt.% therefore did not imply equally strong statistical separation in every material and loading mode.

### 3.4. Fracture-Surface Morphology

#### 3.4.1. Morphology of the As-Received MWCNTs

**Figure 10:**
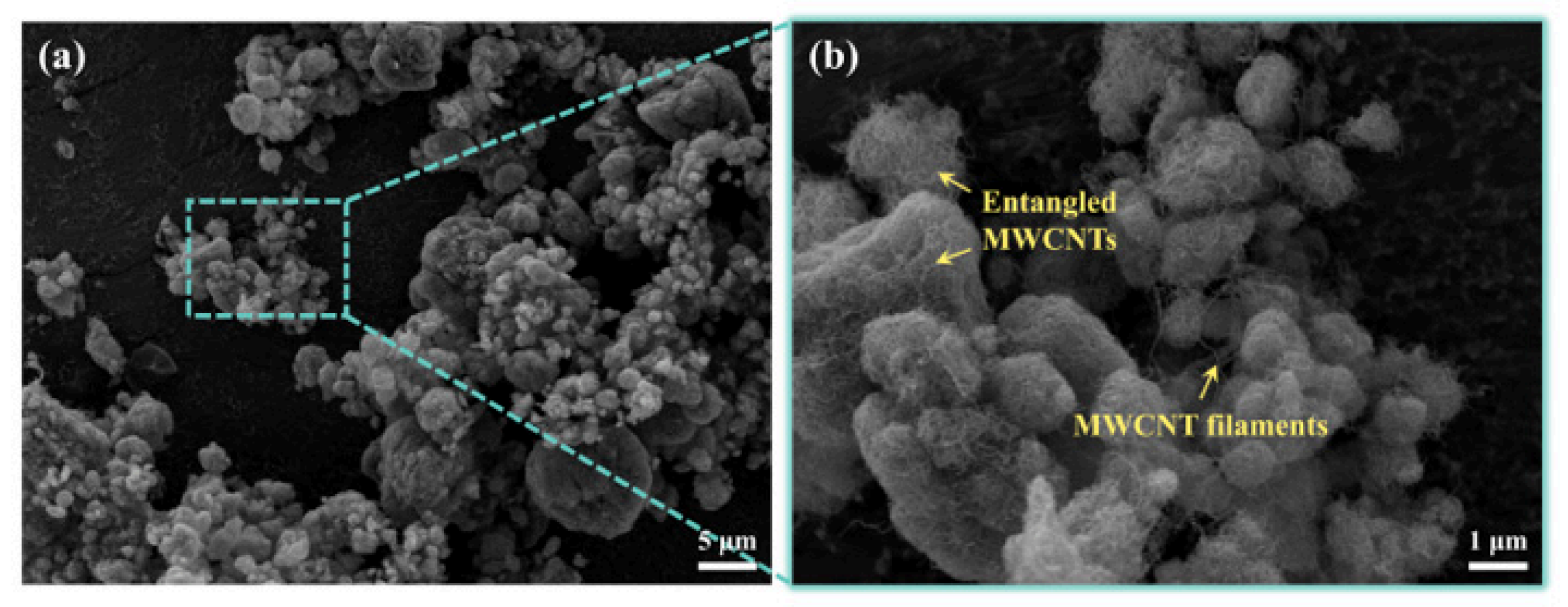
As-received MWCNT powder: (**a**) clusters of different apparent sizes; (**b**) higher-magnification view of the boxed region, showing entangled nanotubes and filament-like features between clusters. Yellow arrows identify representative features. Scale bars: (**a**) 5 μm; (**b**) 1 μm.

Before incorporation into the epoxy, the as-received MWCNT powder exhibited clusters of different apparent sizes (Figure 10a). At higher magnification, the cluster surfaces were covered by densely entangled nanotubes, with filament-like features extending between adjacent clusters (Figure 10b). This morphology established the initial feedstock state for comparison with the exposed filaments and bundle-like features on the composite fracture surfaces.

#### 3.4.2. Fracture Morphology of the Baseline Laminates

**Figure 11:**
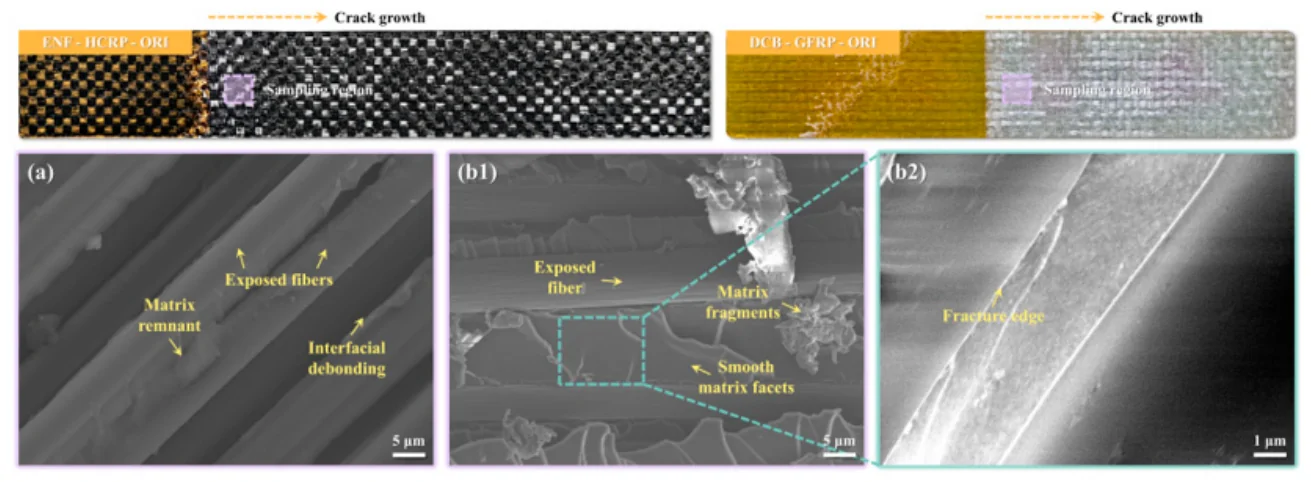
ORI fracture surfaces: (**a**) HCRP after ENF testing, showing exposed fibers, matrix remnants, and fiber–matrix separation; (**b1**) GFRP after DCB testing, showing an exposed fiber, matrix fragments, and smooth matrix facets; and (**b2**) higher-magnification view of the fracture edge. Scale bars: (**a**,**b1**) 5 μm; (**b2**) 1 μm.

**Figure 12:**
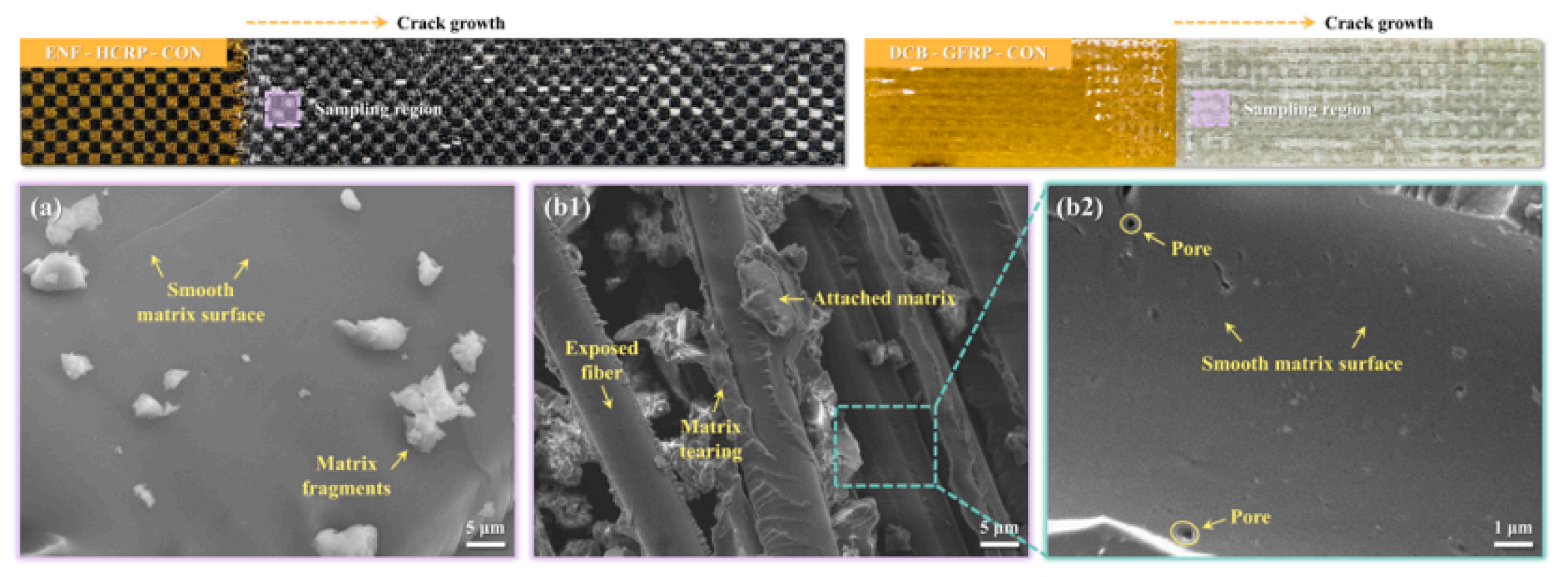
CON fracture surfaces: (**a**) HCRP after ENF testing, showing a smooth matrix region and scattered fragments; (**b1**) GFRP after DCB testing, showing exposed fibers, attached matrix, and localized tearing; and (**b2**) higher-magnification view of the smooth matrix surface with scattered pores. Scale bars: (**a**,**b1**) 5 μm; (**b2**) 1 μm.

GFRP specimens after DCB testing and HCRP specimens after ENF testing were selected for the concentration-dependent comparison because GFRP exhibited the highest absolute Mode I toughness, whereas HCRP showed the largest Mode II improvement at 0.3 wt.%. Figure 11 and Figure 12 show the ORI and CON surfaces, respectively. The upper specimen photographs locate the sampled regions, and the SEM images document matrix- and fiber-scale features at two magnifications. In Figure 11 and Figure 12, purple boxes mark the sampling regions, dashed arrows indicate crack propagation, and yellow annotations identify representative features.

The ORI ENF surface showed exposed fibers, limited matrix remnants, and regions of fiber–matrix separation (Figure 11a). On the ORI DCB surface, exposed fibers coexisted with matrix fragments and comparatively smooth matrix facets (Figure 11(b1)), while a distinct fracture edge was visible at higher magnification (Figure 11(b2)). The prevalence of exposed fibers and smooth regions was consistent with interfacial separation and limited matrix tearing in the examined areas.

The CON ENF surface was dominated by a broad, smooth matrix region with scattered fragments (Figure 12a). The CON DCB surface showed exposed fibers, attached matrix, and localized tearing along matrix-covered regions (Figure 12(b1)). At higher magnification, the matrix remained comparatively smooth and contained scattered pores (Figure 12(b2)). Thus, the neat-epoxy interlayer increased visible matrix participation in some regions without consistently producing extensive tearing, in agreement with the similar ORI and CON toughness values.

#### 3.4.3. Concentration-Dependent Fracture Morphology

**Figure 13:**
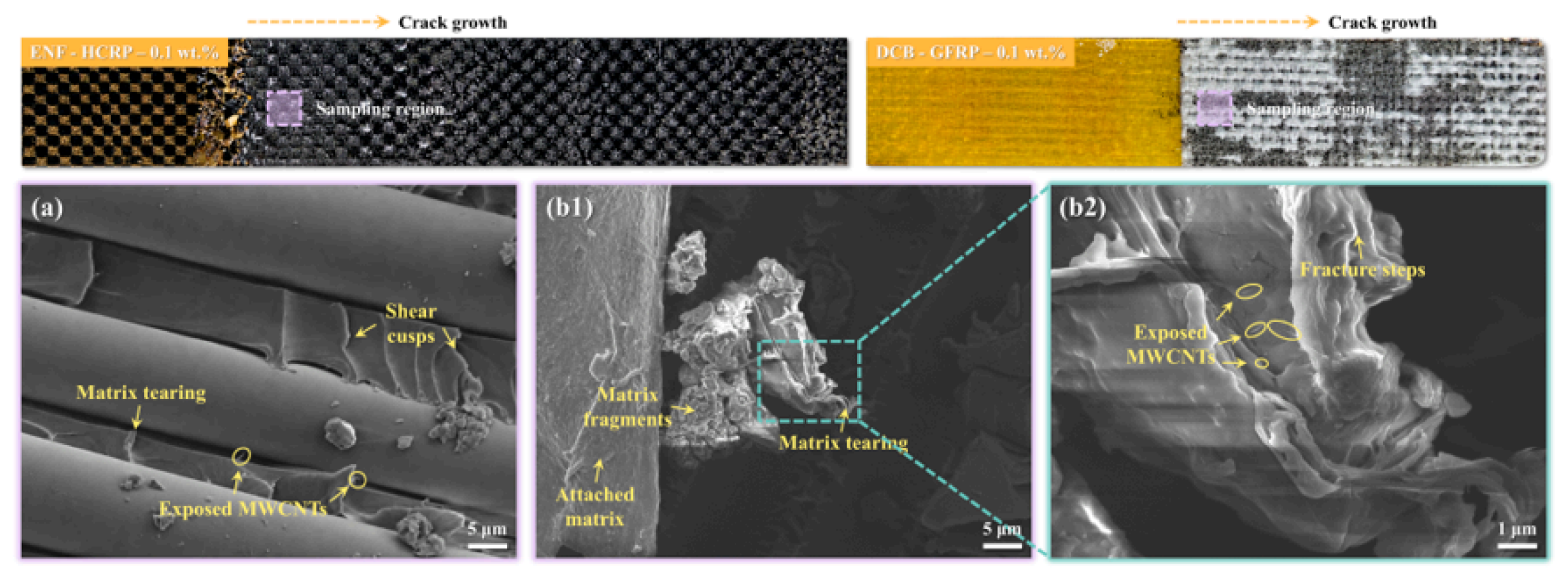
Fracture surfaces at 0.1 wt.% MWCNT: (**a**) ENF-HCRP, showing shear cusps, localized matrix tearing, and fine protrusions; (**b1**) DCB-GFRP, showing attached matrix, fragments, and tearing; and (**b2**) higher-magnification view of fracture steps and locally exposed MWCNTs. Scale bars: (**a**,**b1**) 5 μm; (**b2**) 1 μm.

At 0.1 wt.% MWCNT, the ENF surface contained shear cusps and localized matrix tearing between exposed fibers, together with fine protrusions interpreted as locally exposed MWCNTs (Figure 13a). The DCB surface showed attached matrix, fragments, tearing, and locally exposed nanotubes (Figure 13(b1,b2)). The coexistence of nanotube-related features and matrix fracture indicated that MWCNT–matrix interactions had begun to participate in delamination, although these features remained localized. The corresponding increases relative to CON were 13.1% in *G_s_* for GFRP and 3.4% in *G_IIc_* for HCRP.

**Figure 14:**
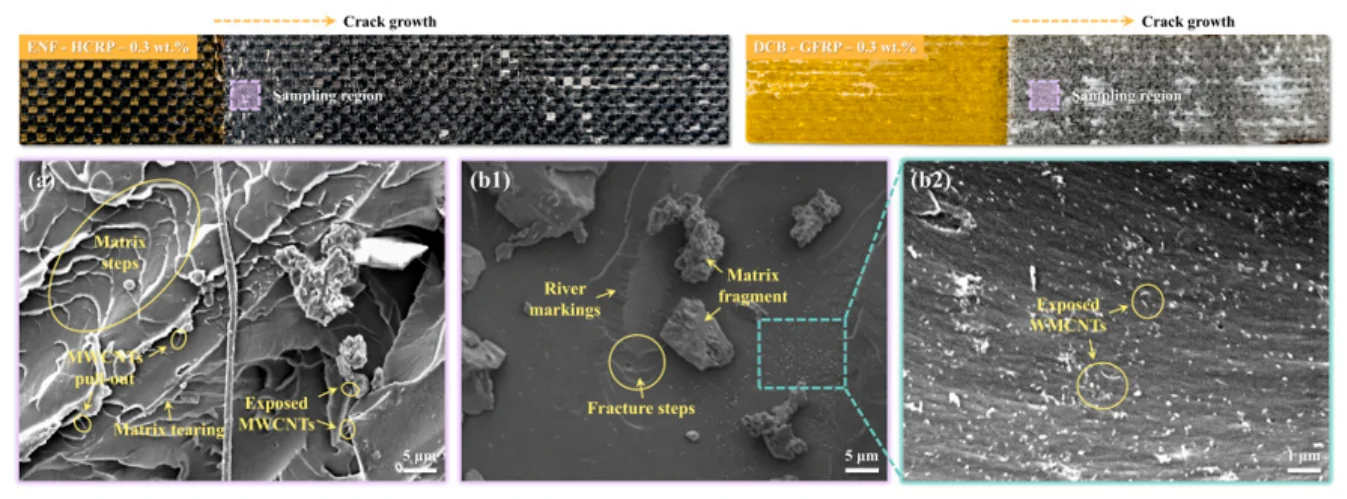
Fracture surfaces at 0.3 wt.% MWCNT: (**a**) ENF-HCRP, showing repeated fracture steps, matrix tearing, and pulled-out-like protrusions; (**b1**) DCB-GFRP, showing river markings, fracture steps, matrix fragments, and fine protrusions; and (**b2**) higher-magnification view of exposed MWCNTs. Scale bars: (**a**,**b1**) 5 μm; (**b2**) 1 μm.

At 0.3 wt.%, the ENF surface exhibited repeated fracture steps, torn matrix edges, and fine projections interpreted as exposed or pulled-out MWCNTs (Figure 14a). The DCB surface showed river markings, fracture steps, matrix fragments, and locally exposed nanotubes (Figure 14(b1,b2)). Compared with 0.1 wt.%, nanotube-related features were more broadly distributed and accompanied more extensive matrix deformation. This concurrent morphology coincided with the highest mean toughness in the representative systems: 38.3% above CON for GFRP under Mode I and 23.1% above CON for HCRP under Mode II.

**Figure 15:**
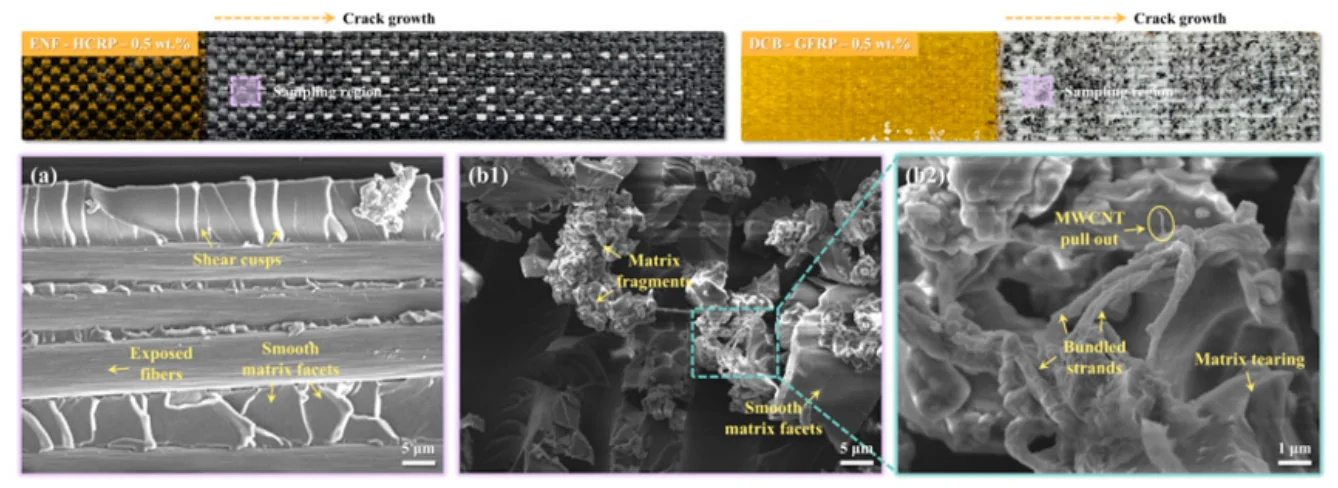
Fracture surfaces at 0.5 wt.% MWCNT: (**a**) ENF-HCRP, showing exposed fibers, shear cusps, and smooth matrix facets; (**b1**) DCB-GFRP, showing matrix fragments and smooth facets; and (**b2**) higher-magnification view of bundled strands, localized matrix tearing, and a pull-out-like protrusion. Scale bars: (**a**,**b1**) 5 μm; (**b2**) 1 μm.

At 0.5 wt.%, the ENF surface combined exposed fibers and shear cusps with comparatively smooth matrix facets (Figure 15a). The DCB surface contained matrix fragments, smooth facets, coarse bundled strands consistent with resin-covered nanotube bundles, localized tearing, and a short pull-out-like protrusion (Figure 15(b1,b2)). The simultaneous presence of localized tearing, a fine protrusion, and coarse strands indicated that nanotube- and matrix-related deformation remained active in some regions, but was less uniformly expressed. Toughness therefore decreased from the 0.3 wt.% peak, while remaining 5.5% and 11.9% above CON for GFRP under Mode I and HCRP under Mode II, respectively. Overall, 0.3 wt.% combined extensive matrix fracture with locally exposed nanotubes, whereas 0.5 wt.% showed larger smooth regions and locally bundled structures, consistent with reduced toughening efficiency.

#### 3.4.4. Cross-Material Fracture Morphology Under Mode II Loading

**Figure 16:**
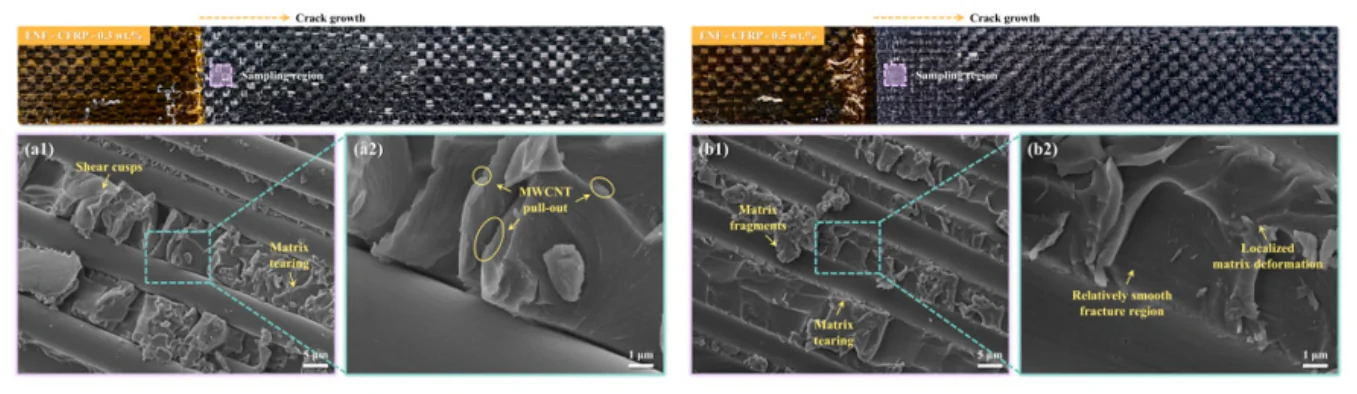
CFRP ENF fracture surfaces at 0.3 wt.% (**a1**,**a2**) and 0.5 wt.% MWCNT (**b1**,**b2**): (**a1**) shear cusps and matrix tearing; (**a2**) pull-out-like features; (**b1**) matrix fragments and tearing; and (**b2**) a comparatively smooth region with localized deformation. Scale bars: (**a1**,**b1**) 5 μm; (**a2**,**b2**) 1 μm.

**Figure 17:**
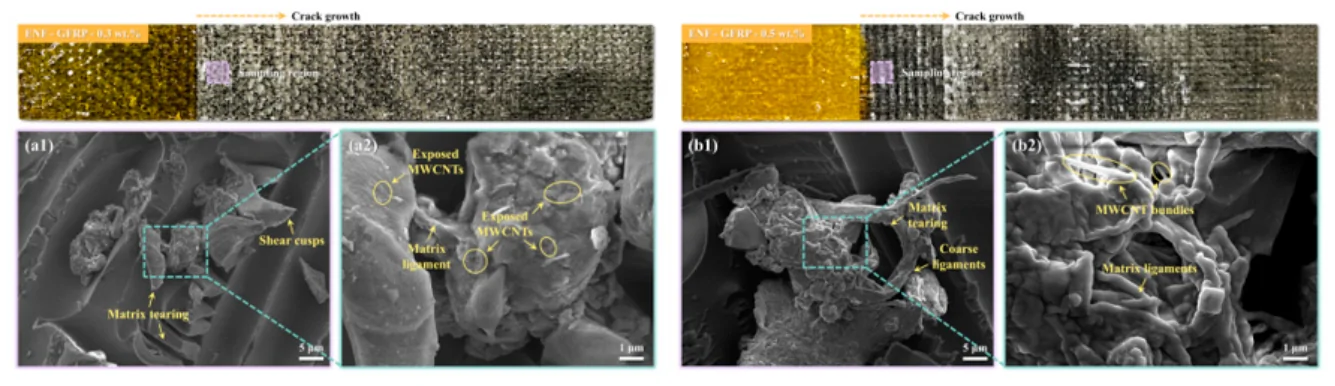
GFRP ENF fracture surfaces at 0.3 wt.% (**a1**,**a2**) and 0.5 wt.% MWCNT (**b1**,**b2**): (**a1**) matrix tearing and shear cusps; (**a2**) nanotube-like features and a matrix ligament; (**b1**) tearing and coarse ligaments; and (**b2**) matrix ligaments and apparent MWCNT bundles. Scale bars: (**a1**,**b1**) 5 μm; (**a2**,**b2**) 1 μm.

**Figure 18:**
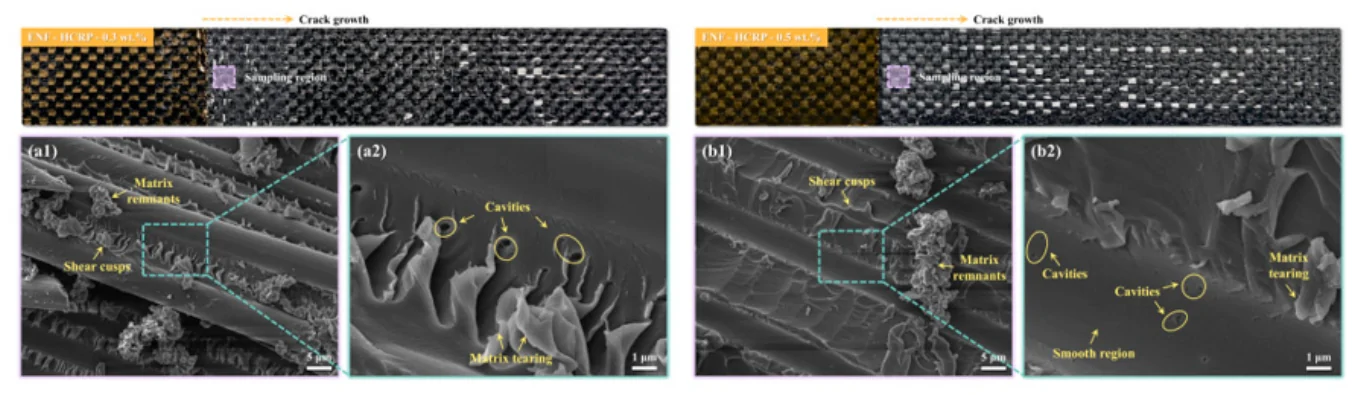
HCRP ENF fracture surfaces at 0.3 wt.% (**a1**,**a2**) and 0.5 wt.% MWCNT (**b1**,**b2**): (**a1**) shear cusps and matrix remnants; (**a2**) cavities adjacent to torn matrix features; (**b1**) shear cusps and matrix remnants; and (**b2**) cavities within a comparatively smooth region with localized tearing. Scale bars: (**a1**,**b1**) 5 μm; (**a2**,**b2**) 1 μm.

**Figure 19:**
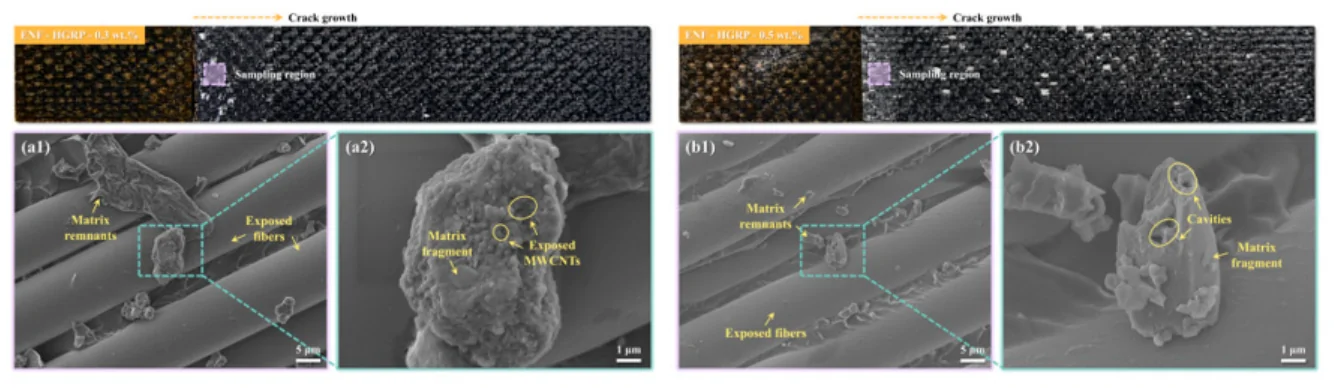
HGRP ENF fracture surfaces at 0.3 wt.% (**a1**,**a2**) and 0.5 wt.% MWCNT (**b1**,**b2**): (**a1**) exposed fibers and matrix remnants; (**a2**) locally exposed nanotube-like features; (**b1**) exposed fibers and matrix remnants; and (**b2**) cavities and attached debris within a matrix fragment. Scale bars: (**a1**,**b1**) 5 μm; (**a2**,**b2**) 1 μm.

Figure 16, Figure 17, Figure 18 and Figure 19 compare ENF fracture surfaces at 0.3 and 0.5 wt.% MWCNT, representing the highest mean Mode II toughness and the subsequent decline in all four systems. ENF specimens were selected because the residual response at 0.5 wt.% differed among materials: CFRP, GFRP, and HCRP remained 9.9%, 12.4%, and 11.9% above CON, respectively, whereas HGRP fell 4.2% below CON. By contrast, Mode I toughness at 0.5 wt.% remained above CON in every system. The comparison therefore focuses on material-dependent Mode II morphologies associated with these different residual responses.

For CFRP, the 0.3 wt.% surface contained shear cusps, torn matrix, and fine protrusions consistent with MWCNT pull-out (Figure 16(a1,a2)). At 0.5 wt.%, matrix fragments and tearing remained visible, but the higher-magnification image showed a comparatively smooth region with localized deformation near its boundaries (Figure 16(b1,b2)). This change accompanied a decrease in mean *G_IIc_* from 2.792 to 2.581 kJ/m^2^, although the 0.5 wt.% value remained 9.9% above CON.

For GFRP, the 0.3 wt.% surface showed matrix tearing, shear cusps, exposed nanotube-like features, and a matrix ligament connecting adjacent regions (Figure 17(a1,a2)). At 0.5 wt.%, coarse ligaments and torn matrix surrounded a matrix-rich fragment, with apparent MWCNT bundles visible at higher magnification (Figure 17(b1,b2)). The corresponding mean *G_IIc_* decreased from 2.755 to 2.629 kJ/m^2^, but remained 12.4% above CON.

For HCRP, both concentrations showed shear cusps and residual matrix along exposed fibers (Figure 18(a1,b1)). At 0.3 wt.%, cavities were adjacent to torn and projecting matrix features, whereas at 0.5 wt.%, they occurred within a comparatively smooth region with localized tearing nearby (Figure 18(a2,b2)). The relevant contrast was therefore the matrix morphology surrounding the cavities rather than their presence alone. Correspondingly, mean *G_IIc_* decreased from 2.859 to 2.600 kJ/m^2^, representing improvements of 23.1% and 11.9% over CON, respectively.

For HGRP, exposed fibers and matrix remnants were present at both concentrations (Figure 19(a1,b1)). The 0.3 wt.% surface contained locally exposed nanotube-like features within a matrix fragment, but the mean *G_IIc_* reached only 2.447 kJ/m^2^, 4.0% above CON (Figure 19(a2)). At 0.5 wt.%, cavities and attached matrix debris were observed within the fragment, and *G_IIc_* decreased to 2.254 kJ/m^2^, 4.2% below CON (Figure 19(b2)). Thus, the common decline from 0.3 to 0.5 wt.% was expressed through material-specific combinations of MWCNT pull-out and localized deformation in CFRP, coarse ligaments and apparent bundles in GFRP, cavity-associated matrix tearing in HCRP, and nanotube-like features or cavities within HGRP matrix fragments. The magnitude of the residual response nevertheless differed markedly among the four systems.

## 4. Discussion

### 4.1. Concentration-Dependent Transition from Toughening to Reduced Efficiency

The absence of a statistically significant ORI-CON difference in any laminate-mode combination suggests that, under the present resin and curing conditions, the neat-epoxy interlayer alone did not measurably alter steady-state Mode I or initiation Mode II toughness. Changes in the MWCNT-modified groups can therefore be associated mainly with nanotube addition rather than with the added resin layer. This conclusion is process-specific: a homogeneous 50 µm epoxy interlayer previously left Mode I toughness nearly unchanged while increasing Mode II initiation and propagation toughness to approximately 1.6 and 3.4 times the noninterleaved values [43], showing that neat-resin effects depend on resin ductility, interlayer geometry, crack location, and loading mode.

All eight laminate-system–fracture-mode combinations reached their highest mean toughness at 0.3 wt.% MWCNT and declined at 0.5 wt.%, but statistical separation was material- and mode-dependent. Significant overall concentration effects occurred in five combinations: GFRP and HGRP under Mode I, and CFRP, GFRP, and HCRP under Mode II. HCRP showed the clearest Mode II peak, with 0.3 wt.% significantly exceeding CON, 0.1 wt.%, and 0.5 wt.%, whereas HGRP showed no statistically detectable Mode II concentration effect. Thus, the common nonmonotonic ordering of the means did not imply equally strong statistical evidence in every system.

At 0.3 wt.%, steady-state Mode I toughness increased by 31.2–51.2% and Mode II initiation toughness increased by 4.0–23.1% relative to CON. These gains were moderate compared with those reported for controlled CNT architectures: magnetically oriented CNTs at 0.3 wt.% produced Mode II improvements of approximately 62.9% for randomly oriented CNTs and 95.2% for aligned CNTs [34]. The concentration associated with maximum toughening also varies with processing route; spray-deposited MWCNT systems have approached maximum toughening near 0.05 wt.% [35], whereas dual-mode toughness in CNT interlayers has peaked near 1 g/m^2^ before declining [36]. Therefore, 0.3 wt.% was the most effective concentration tested for this DMF-assisted ultrasonication and hot-pressing route, not a universal optimum.

### 4.2. Fractographic Evidence for Energy-Dissipation Mechanisms

The fracture surfaces were consistent with three interacting classes of energy dissipation: MWCNT debonding and pull-out with interfacial friction, crack deflection or branching, and matrix deformation. Nanotube extraction dissipates energy through debonding and friction, while nanotube–crack interactions can transfer load and retard opening or sliding. Dispersed nanotubes and their interphases can also divert cracks, increasing path tortuosity and fracture-surface area. Matrix tearing, river markings, fracture steps, and Mode II shear cusps indicate fracture-zone deformation, consistent with reported CNT bridging, pull-out, crack deflection, and matrix shear-yielding mechanisms [8,44].

MWCNT content primarily changed the extent and spatial distribution of these mechanisms. At 0.1 wt.%, localized matrix tearing, fracture steps, shear cusps, and relatively few nanotube-related protrusions indicated spatially restricted MWCNT–matrix participation. At 0.3 wt.%, more broadly distributed nanotube-related features coexisted with pronounced tearing, shear cusps, river markings, and tortuous fracture features. The peak response can therefore be associated with more extensive concurrent activation of pull-out, friction, crack deflection, and matrix deformation rather than with a fundamentally different mechanism.

At 0.5 wt.%, larger smooth regions, coarse bundled or agglomerate-like structures, cavities or pores, and less extensive matrix deformation accompanied the decline in toughness. Local tearing and nanotube-related features remained, indicating reduced rather than eliminated MWCNT-mediated dissipation. Incomplete infiltration of CNT-rich regions and local stiffness mismatch can convert potential bridging sites into stress concentrators or preferential crack paths [8,44], reducing the fraction of nominal nanotube content that participates effectively. A similar sensitivity to interlayer morphology and infiltration has been reported for woven CFRP with CNT-fiber veils, for which Mode I propagation toughness increased with 5–10 μm veils but decreased markedly when veil thickness reached 15 μm [45]. Because the observations were obtained from selected post-fracture regions, they provide local evidence of nanotube-related features, rather than a quantitative measure of MWCNT dispersion throughout the resin or laminate.

### 4.3. Processing Origins of the Dispersion–Agglomeration Balance

The concentration-dependent transition reflects competition between initial dispersion and subsequent resin processing. Ultrasonic cavitation can temporarily disrupt van der Waals-bound MWCNT bundles, while DMF lowers initial viscosity and facilitates suspension. During solvent removal and transfer into epoxy, increasing local nanotube concentration and decreasing intertube spacing can promote reagglomeration, depending on intertube attraction, dispersion stability, and curing conditions [46]. Prolonged ultrasonication may reduce visible agglomerate size but can also shorten nanotubes and decrease their aspect ratio [47], thereby limiting the potential bridging length.

Higher MWCNT content also raises resin viscosity, hindering flow, wetting, air removal, and infiltration of CNT-rich regions. The resulting voids, resin-starved regions, or poorly infiltrated agglomerates can provide low-resistance crack paths [44]. This offers a processing-based explanation for the coexistence of local pull-out with bundled or pore-containing regions at 0.5 wt.% and for the reduced toughening efficiency. Concentration thresholds therefore cannot be transferred directly among manufacturing routes: three-roll milling, spray deposition, and preformed CNT films or veils impose different shear, evaporation, and infiltration conditions, and CNT-film interlayers may introduce weak interfaces when infiltration is incomplete [48]. The present maximum should consequently be regarded as process-specific.

Further optimization should use finer concentration increments around 0.3 wt.% and quantify mixed-resin viscosity, agglomerate-size distribution, residual solvent, void fraction, and interlayer geometry. Scale-up would require renewed validation of dispersion and solvent removal at larger processing scales. Moreover, each configuration comprised one panel and three within-panel specimens; the analysis therefore characterizes within-panel specimen variability, not panel-to-panel manufacturing variability. Independent panel replication is required before a robust processing window can be defined for structural manufacture.

### 4.4. Effects of Fiber System and Fracture Mode on the Toughening Response

The DCB responses reflected both laminate stiffness and fracture processes. CFRP and HCRP, with longitudinal carbon tows, showed shorter displacement ranges than GFRP and HGRP, consistent with greater beam-arm rigidity and lower compliance; the lower modulus and higher failure strain of glass fibers permit larger opening displacements. The response also depends on Mode I resistance and crack-wake mechanisms such as fiber bridging and matrix deformation. GFRP exhibited the highest mean steady-state Mode I toughness under every interlayer condition. GFRP with the same epoxy matrix has shown higher quasi-static Mode II and mixed-mode toughness than CFRP [16], while interlayer efficiency depends on fiber type and interface properties [49]. Glass-fiber sizing, fiber–matrix adhesion, woven interlocking, and resin-pocket geometry may also contribute; therefore, the absolute Mode I ranking cannot be attributed to the MWCNT interlayer alone.

The hybrid laminates differed most clearly under Mode II loading. HCRP showed the largest relative increase and the clearest statistically separated peak at 0.3 wt.%, whereas HGRP showed no significant overall concentration effect. Rotating the same co-woven prepreg by 90° therefore altered interlayer effectiveness. Carbon/glass stacking sequence and precracked-interface location affect Mode I and Mode II fracture [39], while tow orientation can alter initiation toughness, surface roughness, and crack-path selection [41]. Longitudinal carbon tows in HCRP may have increased local rigidity and shear transfer, with stiffness mismatch promoting strain gradients, matrix shear deformation, and nanotube-related friction. In HGRP, compliant longitudinal glass tows may have shifted dissipation toward fabric-scale processes, while crack migration along tow boundaries or resin pockets may have bypassed CNT-rich regions.

Fracture mode further modified toughening efficiency. Mode I favors crack-face bridging, tow pull-out, and progressive crack-wake development, whereas Mode II emphasizes matrix yielding, shear cusps, frictional sliding, and unstable initiation. Ethanolamine-modified CNT films have increased CFRP Mode I toughness by approximately 206% while reducing Mode II toughness by about 8% [33]. Greater CNT-veil thickness likewise does not necessarily improve both modes in parallel because wetting, interlayer thickness, and crack-path selection affect them differently [31]. The common highest mean response at 0.3 wt.% therefore does not imply equal efficiency across fiber architectures or fracture modes.

For weakly responsive configurations such as HGRP under Mode II loading, improvement may require control of nanotube placement or orientation rather than an increase in nominal content. Resin-film processing can control CNT distribution and alignment within interlaminar regions [45], while magnetic alignment can introduce directional through-thickness reinforcement [50]. Such strategies should be evaluated in relation to fiber architecture and the expected delamination mode, because added complexity requires a reproducible toughness gain.

## 5. Conclusions

No statistically significant ORI-CON difference was detected under either fracture mode, indicating that the contribution of the neat-epoxy interlayer alone was not statistically resolved under the present conditions. The 0.3 wt.% MWCNT condition produced the highest mean toughness in all eight laminate-system–fracture-mode combinations, increasing steady-state Mode I toughness by 31.2–51.2% and Mode II initiation toughness by 4.0–23.1% relative to CON. Significant overall concentration effects occurred in five combinations, confirming material- and mode-dependent statistical separation. GFRP had the highest absolute Mode I toughness, HCRP showed the clearest Mode II response at 0.3 wt.%, and HGRP showed no statistically detectable Mode II concentration effect. Thus, 0.3 wt.% was the most effective concentration tested, not a universal optimum.

At 0.3 wt.%, matrix tearing, shear cusps, tortuous features, and distributed nanotube-related pull-out indicated concurrent crack deflection, matrix deformation, and frictional dissipation. At 0.5 wt.%, smooth regions, bundled or agglomerate-like structures, and cavities or pores accompanied reduced efficiency, reflecting competition between MWCNT-mediated dissipation and processing-related defects. The 0.3 wt.% formulation therefore performed best among the tested conditions for this process but should not be regarded as a fully optimized structural design. Further work should refine the concentration and processing window; quantify dispersion, rheology, voids, residual solvent, and interlayer geometry; and examine controlled nanotube placement or alignment. Independent panel replication and validation under mixed-mode, fatigue, impact, and environmental loading are required before structural application.

## Figures and Tables

**Table 1:** Basic properties of the T300 carbon and E-glass reinforcing fibers.

Fibers	Density	Young’s Modulus	Tensile Strength	Strain to Failure
	(kg/m^3^)	(GPa)	(MPa)	(%)
Carbon	1800	230	3530	1.5
Glass	2500	72	2000	2.5

**Table 2:** Steady-state Mode I interlaminar fracture toughness (mean ± SD, *n* = 3) and changes relative to CON for the four laminate systems.

Laminate System	Interlaminar Condition	*G_s_* (kJ/m^2^)	Change Relative to CON
		Mean ± SD	(%)
CFRP	ORI	0.511 ± 0.058	—
	CON	0.510 ± 0.027	—
	0.1 wt.% MWCNT	0.579 ± 0.073	+13.6
	0.3 wt.% MWCNT	0.771 ± 0.103	+51.2
	0.5 wt.% MWCNT	0.529 ± 0.063	+3.8
GFRP	ORI	1.175 ± 0.038	—
	CON	1.152 ± 0.079	—
	0.1 wt.% MWCNT	1.303 ± 0.070	+13.1
	0.3 wt.% MWCNT	1.593 ± 0.016	+38.3
	0.5 wt.% MWCNT	1.215 ± 0.114	+5.5
HCRP	ORI	0.886 ± 0.030	—
	CON	0.838 ± 0.056	—
	0.1 wt.% MWCNT	0.944 ± 0.060	+12.6
	0.3 wt.% MWCNT	1.099 ± 0.077	+31.2
	0.5 wt.% MWCNT	0.894 ± 0.094	+6.7
HGRP	ORI	0.976 ± 0.036	—
	CON	0.965 ± 0.041	—
	0.1 wt.% MWCNT	1.244 ± 0.037	+29.0
	0.3 wt.% MWCNT	1.352 ± 0.066	+40.1
	0.5 wt.% MWCNT	1.139 ± 0.041	+18.1

Percentage changes were calculated from the group means using Equation (5).

## Data Availability

The data presented in this study are available on request from the corresponding author.
